# The glucose transporter 2 regulates CD8^+^ T cell function via environment sensing

**DOI:** 10.1038/s42255-023-00913-9

**Published:** 2023-10-26

**Authors:** Hongmei Fu, Juho Vuononvirta, Silvia Fanti, Fabrizia Bonacina, Antonio D’Amati, Guosu Wang, Thanushiyan Poobalasingam, Maria Fankhaenel, Davide Lucchesi, Rachel Coleby, David Tarussio, Bernard Thorens, Robert J. Hearnden, M. Paula Longhi, Paul Grevitt, Madeeha H. Sheikh, Egle Solito, Susana A. Godinho, Michele Bombardieri, David M. Smith, Dianne Cooper, Asif J. Iqbal, Jeffrey C. Rathmell, Samuel Schaefer, Valle Morales, Katiuscia Bianchi, Giuseppe Danilo Norata, Federica M. Marelli-Berg

**Affiliations:** 1 William Harvey Research Institute, Faculty of Medicine and Dentistry, Queen Mary University of London, London, UK; 2 Department of Pharmacological and Biomolecular Sciences (DisFeB), Università Degli Studi di Milano, Milan, Italy; 3 Section of Anatomical Pathology Department of Precision and Regenerative Medicine, University of Bari Medical School, Bari, Italy; 4 Bart’s Cancer Institute, Faculty of Medicine and Dentistry, Queen Mary University of London, London, UK; 5 Faculty of Biology and Medicine, Center for Integrative Genomics, Génopode Building - UNIL Sorge, University of Lausanne, Lausanne, Switzerland; 6 Discovery Sciences, Innovative Medicines and Early Development Biotech Unit, AstraZeneca, Cambridge, UK; 7 Institute of Cardiovascular Sciences, University of Birmingham, Birmingham, UK; 8 Department of Pathology, Microbiology, and Immunology, Vanderbilt Center for Immunobiology, Vanderbilt University Medical Center, Nashville, TN USA

**Keywords:** Adaptive immunity, Cell biology, Metabolism

## Abstract

T cell activation is associated with a profound and rapid metabolic response to meet increased energy demands for cell division, differentiation and development of effector function. Glucose uptake and engagement of the glycolytic pathway are major checkpoints for this event. Here we show that the low-affinity, concentration-dependent glucose transporter 2 (Glut2) regulates the development of CD8^+^ T cell effector responses in mice by promoting glucose uptake, glycolysis and glucose storage. Expression of Glut2 is modulated by environmental factors including glucose and oxygen availability and extracellular acidification. Glut2 is highly expressed by circulating, recently primed T cells, allowing efficient glucose uptake and storage. In glucose-deprived inflammatory environments, Glut2 becomes downregulated, thus preventing passive loss of intracellular glucose. Mechanistically, Glut2 expression is regulated by a combination of molecular interactions involving hypoxia-inducible factor-1 alpha, galectin-9 and stomatin. Finally, we show that human T cells also rely on this glucose transporter, thus providing a potential target for therapeutic immunomodulation.

## Abstract (web summary)

In this study, Fu et al. provide mechanistic insight into how GLUT2 fine-tunes environmental nutrient sensing with T cell activation, which optimizes metabolic adaptation during acquisition of T cell effector function.

## Main

T cells can adapt metabolic pathways to their specific functions. Quiescent naïve T cells rely primarily on fatty acid oxidation^1^, and following activation they switch to glycolytic energy production and increased biosynthesis, supporting their proliferation. T cell differentiation into specialized subpopulations allows the immune system to optimally respond to pathogen challenges^2^. CD8^+^ T cells proliferate rapidly and differentiate into cytotoxic CD8^+^ T cells, which produce inflammatory cytokines such as interferon (IFN)-γ to target and kill infected and transformed cells^3^. Specific metabolic adaptations associated with distinct T cell subsets have been related to their function in the immune response^4^. CD8^+^ T cell primary and secondary responses are strongly associated with engagement of the glycolytic pathway^5^.

Glucose uptake by T cells is a key metabolic checkpoint, mediated by facilitated diffusion glucose transporters. Glucose transporter family members have distinct amino acid sequences, substrate specificities, kinetic properties and tissue and cellular localizations and are differentially regulated^6,7^. Glucose transporters can be divided into subclasses. Class I facilitative glucose transporters include Glut1, Glut2, Glut3 and Glut4 (refs. ^6,7^).

Glut1 is a high-affinity glucose transporter with a *K*_m_ value for glucose of around 3–7 mM and has been proposed as the primary glucose transporter of T cells^8^. Cell surface expression of Glut1 is nearly undetectable in quiescent T cells, but it becomes strongly upregulated to fuel glycolysis after activation^9–11^. However, studies with Glut1-deficient mice have shown that glycolytic CD8^+^ T cells are less dependent on Glut1 compared to their CD4-expressing counterpart^12^, suggesting the other glucose transporters might support efficient glucose uptake in the CD8^+^ T cell subset.

Glut2 has a uniquely low affinity (*K*_m_ ∼ 17 mM) and high capacity for glucose transport, hence it is most efficient at relatively high glucose concentration^13^. Glut2 is expressed in hepatocytes, absorptive epithelial cells of the intestinal mucosa and kidney, and pancreatic beta cells. In pancreatic beta cells, by virtue of its low affinity, Glut2 can ‘sense’ increases in glucose levels and is required for glucose-stimulated insulin secretion^14^. An important consequence of these properties is that Glut2 can adapt its expression and function in response to environmental changes, such as glucose availability in different body compartments. Although this feature might be advantageous during exposure of recirculating T cells to different microenvironments, a putative role of Glut2 in the immune system has not been investigated. In this study, we show that Glut2 is instrumental to the metabolic regulation of effector function in primed CD8^+^ T cells.

## Results

### Glut2 regulates CD8^+^ T cell metabolism

**Extended Data Fig. 1:**
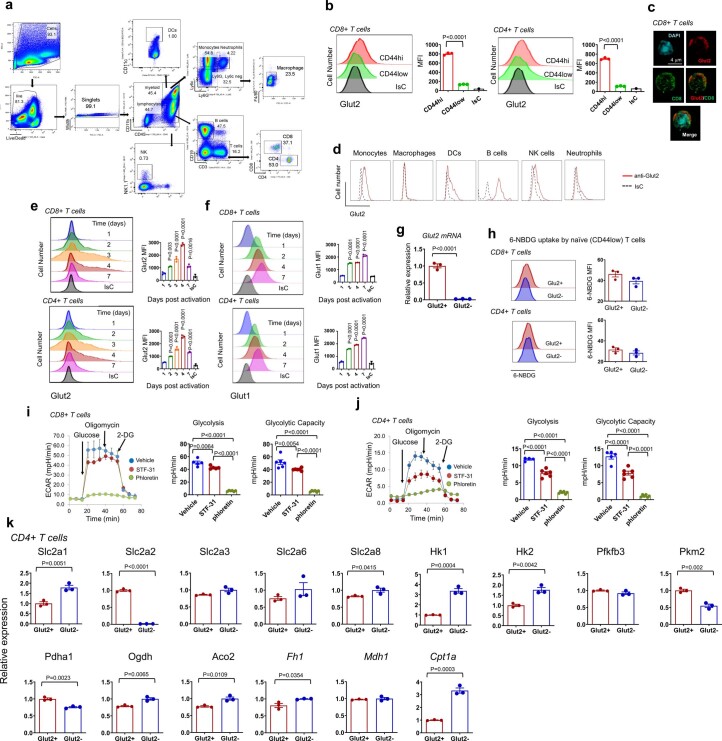
Glut2 expression by immune cells and its effects on T cell metabolism. (**a**) Gating strategy for the identification of immune cell subsets (applies to all Figures and extended data Figures). (**b**) Expression of Glut2 by ex vivo mouse naïve (CD44low) and memory (CD44high) CD8+ and CD4+ T cells was analysed by flow cytometry. Histograms and mean data from a representative experiment (±SD; n = 3, N = 3) are shown. (**c**) Representative confocal microscopy images of DAPI, Glut2 and CD8 expression by antibody-activated CD8 + T cells. (**d**) Representative histograms of Glut2 expression by the indicated immune cell subsets *ex-vivo* (n = 2, N = 2). (**e-f**) Mouse naïve T cells were activated with plate-bound anti-CD3 (1 µg/ml) and anti-CD28 (5 µg/ml) monoclonal antibodies for 1-7 days before analysing surface Glut2 (**e**) and Glut1 (**f**) expression by flow cytometry. Histograms and mean data from a representative experiment (±SD; n = 3, N = 3) are shown. (**g**) T cells were purified from Glut2+ and Glut2- mice and Glut2 transcription was measured by RT-PCR. Gene expression was normalized to housekeeping gene and control Glut2+ set as 1 (±SD; n = 3, N = 3). (**h**) The ability of CD44low T cells to take up the fluorescent glucose analogue 6-NBDG were analysed by flow cytometry. Histograms and mean data from a representative experiment (±SD; n = 3, N = 3) are shown. (**i-j**) Energy metabolism was assessed by extracellular acidification rate (ECAR, mpH/min) in (**i**) activated CD8 + T cells, (**j**) activated CD4+ T cells treated with Glut1 inhibitor (STF-31, 1.25 µM) or Glut2 and Glut1 dual inhibitor (Phloretin, 75 µM) or vehicle. Bar graphs show mean glycolysis and glycolytic capacity (±SD; n = 4-6, N = 2). (**k**) Transcription of the indicated genes in 2-day activated Glut2+ and Glut2- CD4+ T cells was measured by RT-PCR. Gene expression was normalized to tubulin and control Glut2+ set as 1. (±SD; n = 4-6, N = 2). **b, h**, unpaired, two-tailed Student’s t-test; **e-f, i-j**, One-way ANOVA; **g, k**, Kruskal-Wallis test. Source data

A putative role of Glut2 in T cell function was investigated. Glut2 is expressed by mouse memory T cells as well as other immune cells (Extended Data Fig. 1a–d). In mouse naive T cells, Glut2 expression was induced by antibody activation (Extended Data Fig. 1e), peaking 3–4 d after stimulation. Glut1 expression was also increased, peaking 7 d after activation (Extended Data Fig. 1f).

Given its biological function as a glucose transporter, the ability of Glut2 to mediate glucose uptake and T cell metabolic reprogramming following stimulation was investigated by using Glut2-deficient (Glut2^−^) T cells (Extended Data Fig. 1g)^15^.

**Fig. 1:**
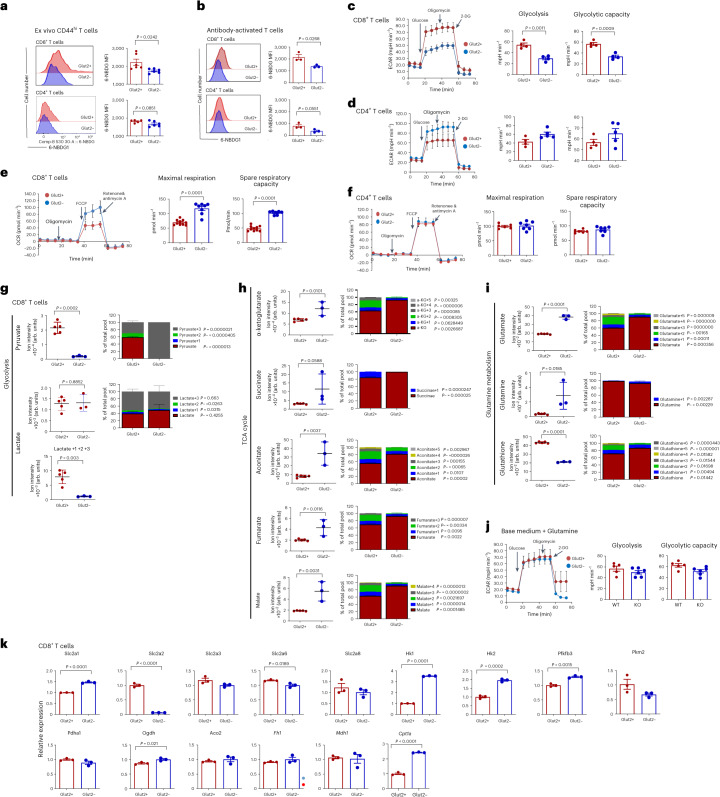
Glut2 affects T cell metabolism. **a**,**b**, 6-NBDG uptake by ex vivo CD44^hi^ (**a**) and 3-d in vitro-activated (**b**) mouse Glut2^+^ and Glut2^−^ CD8^+^ and CD4^+^ T cells was analysed by flow cytometry. Representative histograms and mean data (±s.d.; *n* = 3–6, *N* = 2) are shown. **c**,**d**, ECAR (mpH min^−1^) in 2-d activated mouse Glut2^+^ and Glut2^−^ CD8^+^ and CD4^+^ T cells, respectively. The bar graph shows the mean glycolysis and glycolytic capacity (±s.d., *n* = 3–5, *N* = 2). **e**,**f**, Oxygen consumption rate (OCR; pmol min^−1^) in 2-d activated mouse Glut2^+^ and Glut2^−^ CD8^+^ (**e**) and CD4^+^ (**f**) T cells. The bar graph shows the mean maximal respiration and spare respiratory capacity (±s.d.; *n* = 6–10, *N* = 2). **g**–**i**, 48 h activated mouse Glut2^+^ and Glut2^−^ CD8^+^ T cells were incubated with ^13^C_6_-glucose for 18 h, followed by metabolite extraction for liquid chromatography–mass spectrometry (LC–MS) analysis. Fractional enrichment of ^13^C isotopologues related to glycolysis (**g**), TCA cycle (**h**) and glutamine metabolism (**i**). Column graphs show total levels of each metabolite in the samples on the left-hand side and the proportion of isotopologues of each metabolite indicated by the ‘M + *n*’, which designates the position in the molecule where the ^13^C label is found on the right-hand side. Data are presented as the mean ± s.d. (*n* = 3–5, *N* = 2). **j**, ECAR in 2-d activated mouse Glut2^+^ and Glut2^−^ CD8^+^ T cells supplemented with glutamine (2 mM). The bar graph shows the mean glycolysis and glycolytic capacity (±s.d., *n* = 5–6, *N* = 1). **k**, Transcription of the indicated genes in 2-d activated mouse Glut2^+^ and Glut2^−^ CD8^+^ T cells was measured by PCR with reverse transcription (RT–PCR; *n* = 3, *N* = 2). Gene expression was normalized to housekeeping genes and control Glut2^+^ was set as 1 (mean ± s.d.) **a**–**f**,**j**,**k**, unpaired, two-tailed Student’s *t*-test; **g**–**i**, unpaired, two-tailed Student’s *t*-test, left columns; Mann–Whitney test, right columns. Source data

Uptake of the fluorescent glucose analogue 6-NBDG was significantly reduced in ex vivo Glut2^−^ memory, and in vitro-activated CD8^+^ but not CD4^+^ T cells (Fig. 1a,b). Baseline glucose uptake by either naive CD4^+^ or CD8^+^ T cells was not affected by lack of Glut2 expression (Extended Data Fig. 1h).

We then investigated Glut2-mediated glucose utilization by activated T cells via metabolic fluxometry. As shown in Fig. 1c,d, engagement of the glycolytic pathway after 3-d antibody activation was significantly reduced in Glut2^−^ CD8^+^ T cells, while it was slightly increased in Glut2^−^ CD4^+^ T cells. Activated Glut2^−^ CD8^+^ T cells displayed increased oxidative phosphorylation (OxPhos; Fig. 1e), possibly as a compensatory response to the reduced energy supply, while mitochondrial respiration was similar in CD4^+^ T cells irrespective of Glut2 expression (Fig. 1f). Additionally, the Glut1-selective inhibitor STF-31 and the dual Glut1 and Glut2 inhibitor phloretin^16^ were used to address the relative contribution of these transporters to the glycolysis in activated wild-type (WT) CD4^+^ and CD8^+^ T cells. Both CD8^+^ and CD4^+^ T cells exposed to STF-31 displayed a significantly reduced glycolytic activity, and this effect was more pronounced in the CD4^+^ subset (Extended Data Fig. 1i,j). Exposure to phloretin dramatically reduced the glycolytic flux in both CD4^+^ and CD8^+^ T cells, suggesting that both glucose transporters are required to sustain glucose metabolism in CD4^+^ and CD8^+^ T cells. Glut1 is instrumental to CD4^+^ glucose metabolism, but at least in part redundant in CD8^+^ T cells. However, in the absence of Glut2 expression, Glut1 contributes to sustaining CD8^+^ T cell glycolytic activity.

Based on these observations, we used ^13^C_6_-glucose labelling to study metabolic fluxes in activated Glut2^+^ and Glut2^−^ CD8^+^ T cells. As shown in Fig. 1g, induction of glycolysis was severely impaired in Glut2^−^ CD8^+^ T cells as indicated by decreased pyruvate labelling. Similar levels of pyruvate-3 indicate that all ^13^C_6_-glucose uptake is metabolized to pyruvate. In addition, despite similar levels of unlabelled lactate, the fraction of labelled lactate was significantly lower compared to that of Glut2^+^ T cells.

Glut2^−^ CD8^+^ T cells also displayed a more active tricarboxylic acid (TCA) cycle as suggested by the presence of more metabolites. However, like for lactate, the fraction of TCA metabolites labelled from glucose was reduced compared to that observed in Glut2^+^ T cells (Fig. 1h), suggesting that the increase of these metabolites in Glut2^−^ T cells was from a source different than ^13^C_6_-glucose.

We also detected an increase in glutamine and glutamate accompanied by a decrease in glutathione in Glut2^−^ T cells. The observed increase in unlabelled metabolite fractions might indicate increased glutamine uptake (as glutamine is present in the medium during labelling with ^13^C_6_-glucose) and a decreased generation of these metabolites from glucose. Glutamine can be used to replenish TCA cycle intermediates (anaplerosis) and to provide a robust source of NADPH production, resulting in its catabolism to lactate^17^. This effect likely explains the similar levels of unlabelled lactate produced by Glut2^+^ and Glut2^−^ CD8^+^ T cells. In support of this hypothesis, addition of glutamine in the Seahorse base medium (which does not contain glutamine) increased the extracellular acidification rate (ECAR) of Glut2^−^ CD8^+^ T cells to the same levels of Glut2^+^CD8^+^ T cells (Fig. 1j).

Thus, increased glutamine uptake and glutamate generation might account for the increase of unlabelled TCA metabolites, including α-ketoglutarate and enhanced OxPhos in Glut2^−^ T cells. This compensation might come at the cost of reduced glutathione production.

We then analysed the expression of genes encoding metabolic enzymes by activated Glut2-deficient T cells. Glut2^−^ CD8^+^ T cells upregulated several genes involved in the glycolytic pathway (Fig. 1k). Among the genes encoding enzymes involved in the TCA cycle, Glut2^−^ CD8^+^ T cells only increased expression of Ogdh (encoding α-ketoglutarate-dehydrogenase), in line with the possibility that glutamine might feed into the TCA cycle in these T cells. In addition, transcription of the gene encoding CPT1a, an enzyme essential for the transport of fatty acids in the mitochondria, was also significantly upregulated.

Compared to their CD8^+^ counterparts, Glut2^−^ CD4^+^ T cells also upregulated transcription of a larger number of genes encoding TCA enzymes and glycolytic enzymes (Extended Data Fig. 1k).

These data indicate that both Glut2^−^ CD8^+^ and CD4^+^ T cells undergo a compensatory transcriptional programme capable of correcting metabolic pathways only in the CD4^+^ T cell subset. The lack of TCA gene upregulation, except Ogdh, by Glut2^−^ CD8^+^ T cells might reflect a switch to glutamine utilization.

### Glut2 regulates CD8^+^ T cell function

**Extended Data Fig. 2:**
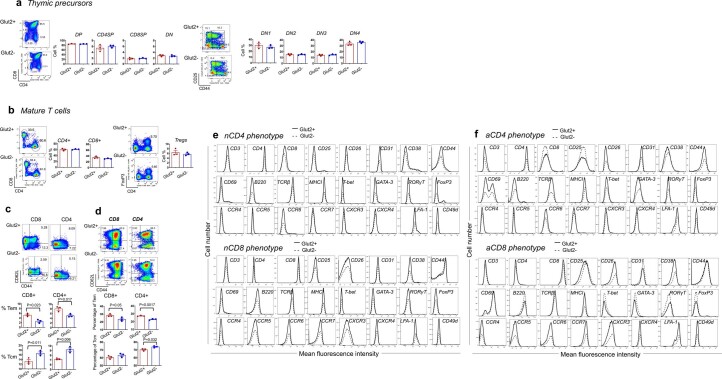
Glut 2 expression and function in T cells. (**a**) Live and single cells from thymi of Glut2+and Glut2-chimera mice were analysed by flow cytometry. Representative dot-plots (left) and mean percentage of double-positive, CD4-single-positive, CD8-single-positive, and double-negative subsets (right) are shown (±SD; n = 3, N = 1). (**b**) T cells from the spleen and lymph nodes of Glut2+and Glut2-mice were analysed for expression of CD3, CD4, CD8, and FoxP3. Representative dot-plots (left) and mean percentage of CD4, CD8, and regulatory (Treg) subsets (right) are shown (±SD; n = 3, N = 1). (**c-d)** CD8+ and CD4+ effector memory T cells (Tem) and central memory T cells (Tcm) in ex-vivo T cells (**c**) and antibody-stimulated (6 days) naïve T cells (**d**) from Glut2+ and Glut2-. Representative dot-plots are shown on the top panel. The percentage of Tem and Tcm cells are shown on the bottom panel (±SD; n = 3, N = 1). Unpaired, two-tailed Student’s t-test. (**e-f**) Expression of the indicated molecules by naive (**e**) and antibody-activated (6 days) (**f**) CD4+ and CD8 + T cells was analysed by flow cytometry (n = 3). Source data

To explore the physiological relevance of Glut2 expression by T cells, we generated Glut2^+^ or Glut2^−^ bone marrow (BM) chimeras. A full characterization of T cells from these mice is provided in Extended Data Fig. 2.

**Fig. 2:**
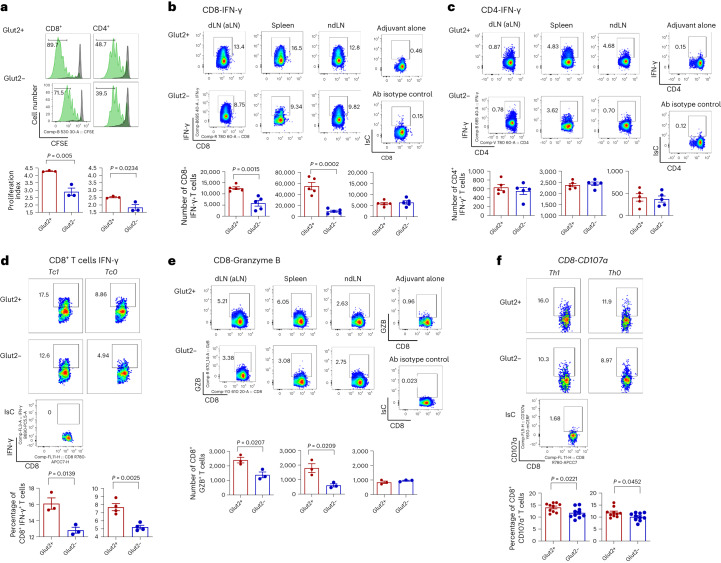
Glut2 contributes to CD8^+^ T cell function. **a**, Mouse naive Glut2^+^ and Glut2^−^ CD8^+^ and CD4^+^ T cells were labelled with CFSE (5 μM) and stimulated with plate-bound anti-CD3 (1 µg ml^−1^) and anti-CD28 (5 µg ml^−1^) monoclonal antibodies for 3 d. Proliferation was assessed by flow cytometry. Histograms (grey indicates unstimulated cells) and mean data from a representative experiment (*n* = 3, *N* = 2) ± s.d. are shown. **b**,**c**, Glut2^+^ and Glut2^−^ chimera mice were primed and boosted (7 d apart) with IP injection of 750 µg ovalbumin protein plus 50 µg poly(I:C) adjuvant or adjuvant alone. After 7 d, T cells were separately harvested from mesenteric lymph nodes (dLN), inguinal and axillary (ndLNs) and the spleen. Expression of IFN-γ was assessed by flow cytometry. Representative dot plots are shown. Control injection with adjuvant alone and staining with an isotype-matched control antibody are shown on the right-hand side of each set. The mean number of IFN-γ^+^ cells from a representative experiment (*n* = 3, *N* = 2) is shown (±s.d.). Percentages are shown. **d**, Production of IFN-γ by mouse Glut2^+^ and Glut2^−^ CD8^+^ T cells that were antibody activated in Tc-1 or Tc-0 polarizing conditions (Methods) was measured by flow cytometry. *n* = 3–4, *N* = 2. **e**, Production of granzyme B by primed (**b**) mouse Glut2^+^ and Glut2^−^ CD8^+^ T cells from a representative experiment (±s.d.; *n* = 3, *N* = 2) is shown. Percentages are shown. **f**, T cells from Glut2^+^ and Glut2-deficient mice were activated by plate-bound CD3/CD28 antibodies, and differentiated toward Th0 and Th1 cells. CD107α was assessed by flow cytometry. Representative dot plots and histograms are shown (±s.d.; *n* = 10, *N* = 2). **a**–**f**, unpaired, two-tailed Student’s *t*-test. Source data

Proliferation of antibody-stimulated naive Glut2^−^ T cells was impaired particularly in the CD8^+^ subset (Fig. 2a). Further, differentiation of Glut2^−^ T cells into the effector memory subset (T_em_) both after ex vivo stimulation (Extended Data Fig. 2c) and after in vitro stimulation (Extended Data Fig. 2d) was reduced.

**Extended Data Fig. 3:**
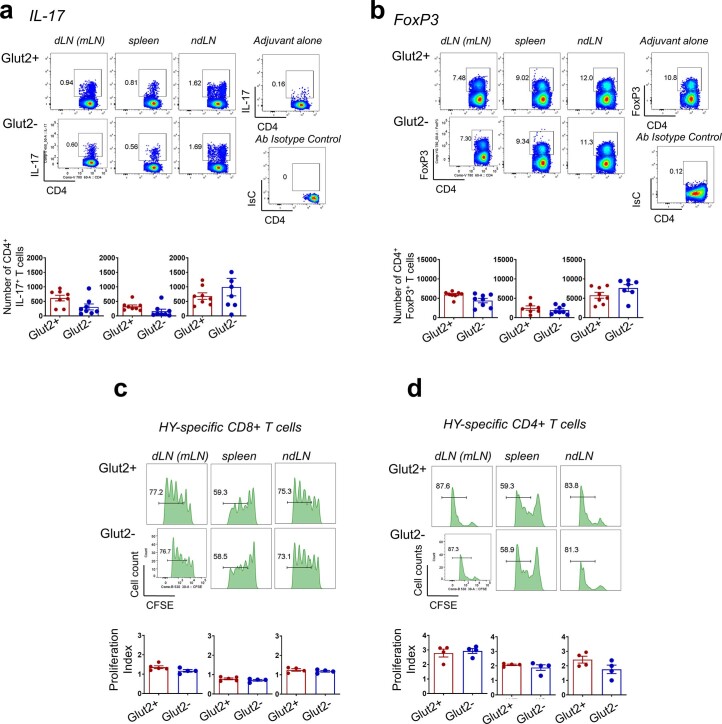
Cytokine production by Glut2- primed T cells and function of Glut2- antigen presenting cells. (**a-b**) Glut2+ or Glut2- chimera mice were primed and boosted (7 days apart) with intra-peritoneal (IP) injection of 750 µg ovalbumin protein plus 50 µg Poly(I:C) adjuvant or adjuvant alone. After 7 days, T cells were separately harvested from mesenteric lymph nodes (draining LN, dLN), inguinal and axillary (non-draining LNs, ndLN) and the spleen. Expression of IL-17 (**a**) and FoxP3 (**b**) were assessed by flow cytometry. Representative dot plots are shown. Staining with an isotype-matched control antibody and adjuvant control alone are shown on the right-hand side of each set of dot plots. The mean number of IL-17+ and FoxP3+ from three independent experiments of identical design is shown on the bottom panel (±SD, n = 8, N = 2). (**c-d**) Effect of Glut2 on APCs. T cells from Mata Hari mice (10^7^/mouse) and Marilyn mice (10^7^/mouse) were labelled with CFSE (5 μM) and injected intravenously into Glut2+ and Glut2- female recipients. Twenty-four hours later, recipient mice received male splenocytes. Five days after immunization, T cells were separately harvested from mesenteric LN (draining LN, dLN), spleen, and inguinal and axillary (non-draining LNs, ndLN). Marilyn T cells were identified as CD4 + Vb6 + CD45.2 + , and donor Mata Hari T cells were identified as CD8 + Vb8.3 + CD45.2 + . CFSE dilution was assessed by flow cytometry. The proliferation index is shown (n = 4, N = 2). Source data

Glut2^+^ and Glut2^−^ chimeric mice were primed and boosted 7 d later with ovalbumin plus poly(I:C) adjuvant by intraperitoneal (IP) injection, or adjuvant alone as a control. A week after booster immunization, T cells were separately harvested from draining lymph nodes (dLNs), non-draining lymph nodes (ndLNs) and the spleen. Production of IFN-γ by activated Glut2^−^ CD8^+^ but not CD4^+^ T cells was reduced (Fig. 2b,c) compared to their Glut2^+^ counterparts. It is well established that efficient engagement of the glycolytic pathway promotes IFN-γ production via post-transcriptional and epigenetic mechanisms in T cells^18,19^. To confirm that loss of Glut2 expression led to a decrease of IFN-γ by cell-intrinsic mechanisms, Glut2^+^ and Glut2^−^ CD8^+^ T cells were activated in Tc-1-polarizing conditions. As shown in Fig. 2d, Tc-1-induced and even non-polarized (Tc-0) Glut2^−^ CD8^+^ T cells displayed a significant defect in IFN-γ production. In addition, production of granzyme B and expression of the degranulation marker CD107α were significantly decreased in Glut2^−^ CD8^+^ T cells (Fig. 2e,f). No difference was observed in the expression of interleukin (IL)-17 and Foxp3 (Extended Data Fig. 3a,b).

**Fig. 3:**
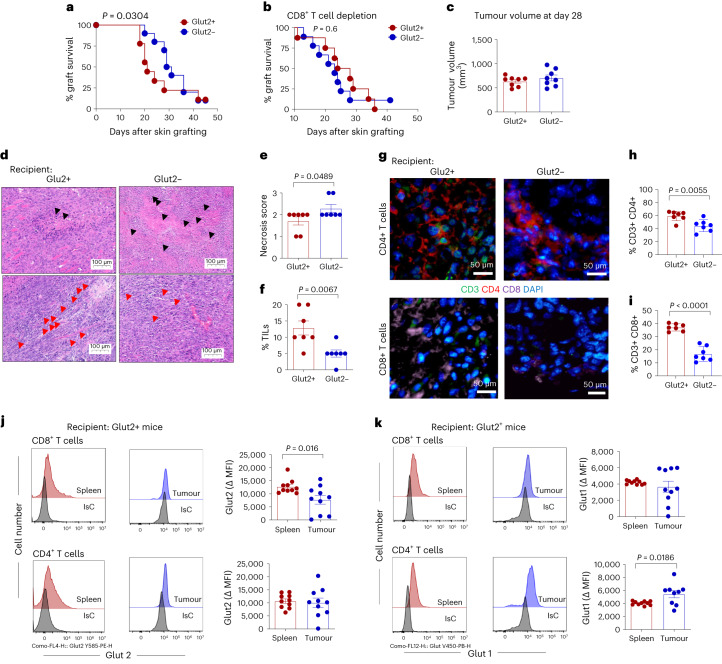
Glut2 is required for optimal CD8^+^ T cell-mediated anti-allograft and anti-tumour immunity. **a**,**b**, Glut2^+^ or Glut2^−^ female mice received skin grafts from female B6.Kd donors, to avoid confounders due to anti-HY responses (**a**). CD8^+^ T cell depletion was achieved by IP injection of 200 μg anti-CD8 at days –1 and 1 (**b**). *P* values of the mean time of rejection are indicated within the graphs (*n* = 8–10, *N* = 1). **c**, EO771 cells were injected into the mammary glands of female Glut2^+^ or Glut2^−^ C57BL/6J mice (*n* = 10). The mean values of tumour volume (mm^3^) are reported (±s.d., *n* = 10, *N* = 1). **d**–**f**, Tumour tissue was embedded in paraffin for H&E staining (**d**) for assessment of necrosis score (**e**) and the percentage of TILs (**f**) (±s.d., *n* = 6, *N* = 1). **g**–**i**, Immunofluorescence staining of CD3, CD4, CD8 and DAPI in OCT-embedded tumour sections was analysed and quantified for the percentage of CD3^+^CD4^+^ T cells (**h**), and the percentage of CD3^+^CD8^+^ T cells (**i**) (±s.d., *n* = 7, *N* = 1). **j**,**k**, T cells from tumour and spleen were assessed for surface expression of Glut2 (**j**) and Glut1 (**k**) by flow cytometry. Delta changes of MFI in Glut2 and Glut1 expression (±s.d.; *n* = 10, *N* = 1) were determined by subtraction of isotype control from antibody staining. **a**,**b**, log-rank (Mantel–Cox) test; **c**–**k**, unpaired, two-tailed Student’s *t*-test. Source data

We subsequently focused on Glut2^−^ CD8^+^ T cell effector function by grafting major histocompatibility complex (MHC) class I molecule-mismatched B6.Kd^20^ skin onto Glut2^+^ and Glut2^−^ chimeric mice. As shown in Fig. 3a, B6.Kd skin rejection was significantly delayed in Glut2^−^ BM chimeras. Antibody-mediated CD8^+^ T cell depletion before grafting resulted in similar rejection kinetics in Glut2^+^ and Glut2^−^ mice (Fig. 3b), further supporting a prominent role of Glut2 in CD8^+^ T cell effector response.

To determine the effect of Glut2 deficiency on antigen-presenting cells, male H-Y antigen-specific T cell antigen receptor (TCR)-transgenic CD4^+^ (Marilyn) and CD8^+^ (Mata Hari (MH)) T cells^21,22^ were labelled with CFSE and adoptively transferred into Glut2^+^ and Glut2^−^ female recipients. Twenty-four hours later, recipient mice received male splenocytes intraperitoneally. Five days after immunization, T cells were separately harvested from dLNs, ndLNs and the spleen, and TCR-transgenic T cell proliferation was assessed. Both male-specific CD4^+^ and CD8^+^ T cells proliferated equally in either Glut2-competent or Glut2-deficient recipients (Extended Data Fig. 3c,d).

Glut2 contribution to anti-tumour immunity was also investigated. As a model, we used orthotopic implantation of the tumour line EO771 (derived from spontaneous breast cancer of C57BL/6J mice) whose growth is efficiently controlled by CD8^+^ T cells^23^, in Glut2^+^ and Glut2^−^ BM chimeras. Tumour growth was monitored for 28 d (the limit allowed by our Home Office License). At this time point, tumour volume did not significantly differ between recipients (Fig. 3c). However, when the tumour tissue was analysed, significantly larger areas of necrosis accompanied by reduced tumour-infiltrating lymphocytes (TILs), both indicators of tumour progression, were observed in Glut2^−^ recipients (Fig. 3d–f). Immunofluorescence staining revealed a small but significant decrease of CD4^+^ T cell infiltration, which was substantial in the CD8^+^ subset (Fig. 3g–i). Expression levels of Glut2 and Glut1 by T cells from TILs and splenocytes from tumour-bearing WT BM chimeras were analysed by flow cytometry. As shown in Fig. 3j,k, Glut2 expression was significantly decreased in CD8^+^ but not CD4^+^ TILs. Conversely, Glut1 expression was significantly increased in CD4^+^ but not CD8^+^ TILs, suggesting that expression of Glut1 and Glut2 are differentially regulated by environmental factors in CD4^+^ and CD8^+^ T cells.

### Glut2 expression by T cells is modulated by glucose, extracellular pH and oxygen availability

In pancreatic beta cells, Glut2 expression is regulated by micro-environmental changes^24^. As memory T cells recirculate through vascular and tissue sites where glucose availability, acidification and oxygen tension differ, such as blood and inflammatory sites, the relative effects of these variables on Glut2 expression and function were investigated.

**Fig. 4:**
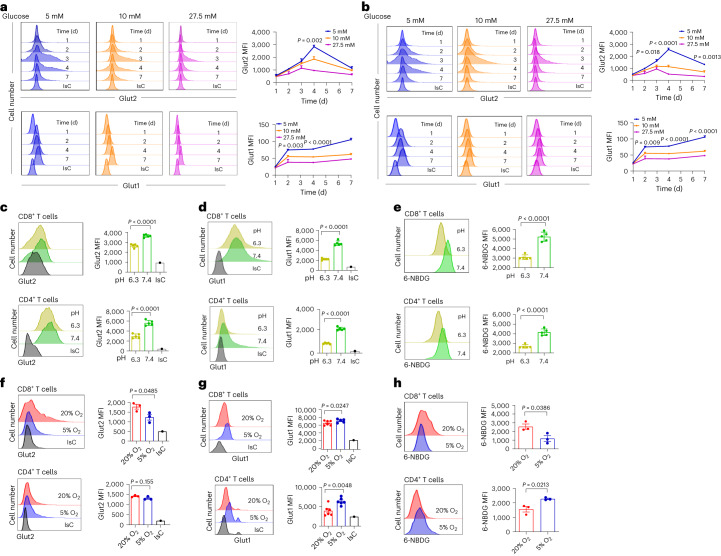
Regulation of Glut expression by environmental factors. **a**,**b**, Mouse naive T cells were activated by antibodies in medium containing different concentrations of glucose for 7 d before assessing surface Glut2 and Glut1 expression by CD8^+^ (**a**) and CD4^+^ (**b**) T cells by flow cytometry. Histograms and mean data (±s.d.) from a representative experiment (*n* = 3, *N* = 3) are shown. **c**–**e**, Mouse T cells were activated by antibodies in medium with a pH of either 6.3 or 7.4 for 3 d before analysing surface expression of Glut2 (**c**), Glut1 (**d**) (*n* = 5, *N* = 3) and 6-NBDG uptake (**e**) (*n* = 3, *N* = 3) by flow cytometry. Histograms and mean data (±s.d.) from a representative experiment are shown. **f**–**h**, Mouse naive T cells were activated by antibodies for 2 d and subsequently maintained in high (20%) or low (5%) oxygen concentrations for the last 24 h of culture (*n* = 5–6, *N* = 3). Surface expression of Glut2 (**f**), Glut1 (**g**) and 6-NBDG uptake (**h**) (*n* = 3, *N* = 3) were analysed by flow cytometry. Histograms and mean data (±s.d.) from a representative experiment are shown. **a**,**b**, one-way analysis of variance (ANOVA); **c–h**, unpaired, two-tailed Student’s *t*-test. Source data

First, we found that activation-induced increases in Glut2 and Glut1 expression by CD8^+^ (Fig. 4a) and CD4^+^ T cells (Fig. 4b) were significantly blunted by glucose concentrations above 5 mM.

To evaluate the effect of extracellular acidification on Glut2 expression, T cells were activated by antibodies in medium at pH 7.4 or pH 6.3. As shown in Fig. 4c–e, a decrease in pH led to a substantial reduction in the expression of both Glut2 and Glut1 and 6-NBDG uptake by both CD8^+^ and CD4^+^ T cells.

Finally, T cells were activated by antibodies for 2 d and subsequently maintained in high (20%) or low (5%) oxygen concentrations for the last 24 h of culture^25^. As shown in Fig. 4f, Glut2 expression by CD8^+^ T cells was significantly reduced in hypoxic conditions. Glut2 expression by CD4^+^ T cells did not change in different oxygen concentrations. Conversely, Glut1 expression was upregulated by CD4^+^ and only marginally by CD8^+^ T cells at 5% oxygen (Fig. 4g). Accordingly, 6-NBDG uptake by CD8^+^ T cells was increased in high oxygen cultures, while CD4^+^ T cells enhanced 6-NBDG uptake in low oxygen conditions (Fig. 4h).

Overall, these data show that expression of both Glut1 and Glut2 by T cells is dictated by environmental cues and is regulated by glucose concentration and extracellular acidification in the same manner. However, Glut2 expression is directly proportional to oxygen availability in CD8^+^ T cells, while a decrease in oxygen leads to an increase in Glut1 expression in both CD4^+^ and CD8^+^ T cells, suggesting that these transporters can be differentially regulated.

### Glut2 expression is regulated by HIF-1α via galectin-9

**Fig. 5:**
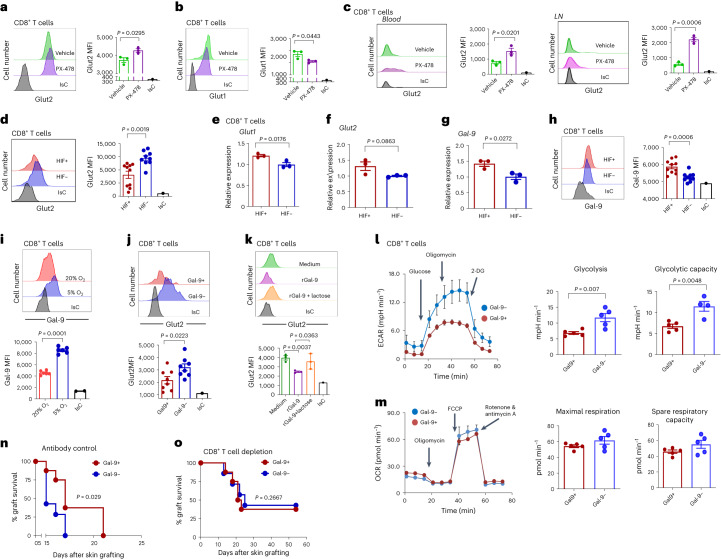
Regulation of Glut2 expression by HIF-1α and galectin-9. **a**,**b**, Mouse naive CD8^+^ T cells were activated by antibodies in the presence or absence of the HIF-1α selective inhibitor PX-478 (20 µM) before analysing surface expression of Glut2 (**a**) and Glut1 (**b**). Histograms and mean data ± s.d. from a representative experiment (*n* = 3, *N* = 3) are shown. **c**, C57BL/6J mice received an IP injection of PX-478 (20 mg per kg body weight) for 3 d. Representative histograms and mean data (±s.d.) of Glut2 expression by CD8^+^ T cells are shown (*n* = 3, *N* = 2). **d**, Glut2 expression by mouse HIF^+^ or HIF^+^ CD44^hi^ CD8^+^ T cells. Representative histograms and mean data (±s.d.) are shown (*n* = 3, *N* = 3). **e**–**g**, Transcription of Glut1 (**e**), Glut2 (**f**) and Gal-9 (**g**) by mouse HIF^+^ or HIF^−^ CD44^hi^ CD8^+^ T cells measured by RT–PCR. Gene expression was normalized to the housekeeping gene tubulin. Control HIF^−^ was set as 1. Error bars show the s.d. (*n* = 3, *N* = 3). **h**, Surface expression of Gal-9 by mouse HIF^+^ and HIF^+^ CD8^+^ T cells. Histograms and mean data (±s.d.) from a representative experiment are shown (*n* = 9–10, *N* = 2). **i**, Mouse naive CD8^+^ T cells were activated by antibodies for 2 d and subsequently maintained in high (20%) or low (5%) oxygen incubators for a further 24 h. Gal-9 surface expression was then analysed. Histograms and mean data (±s.d.) from a representative experiment are shown (*n* = 3, *N* = 2). **j**, Glut2 expression by mouse Gal-9^+^ or Gal-9^−^ CD44^hi^ T cells. Histograms and mean data (±s.d.) from a representative experiment are shown (*n* = 6, *N* = 2). **k**, Mouse naive T cells were activated by antibodies in the presence or absence of recombinant Gal-9 (30 nM) with or without the Gal-9 competitive inhibitor lactose (30 mM) before analysing surface expression of Glut2. Histograms and mean data (±s.d.) from a representative experiment (*n* = 3, *N* = 2). **l**,**m**, ECAR and OCR by antibody-activated mouse Gal-9^+^ and Gal-9^−^CD8^+^ T cells. **l**,**m**, Mean glycolysis and glycolytic capacity (±s.d.; *n* = 5, *N* = 2) (**l**) and mean maximal respiration and spare respiratory capacity (**m**) are shown (±s.d.; *n* = 5, *N* = 2). **n**,**o**, Gal-9^+^ and Gal-9^−^ mice received skin grafts from BL/6.Kd donors (**n**). CD8^+^ T cells were depleted by IP injection of 200 μg anti-CD8 at days –1 and +1 (**o**) (*n* = 7–8, *N* = 1). **a**–**d**,**e**–**j**,**l**,**m**, unpaired two-tailed Student’s *t*-test; **k**, one-way ANOVA; **n**,**o**, log-rank (Mantel–Cox) test. Source data

**Extended Data Fig. 4:**
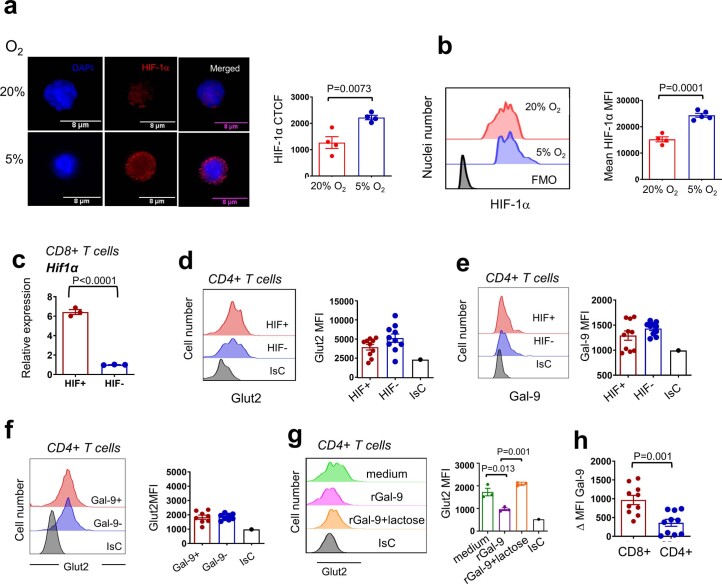
Regulation of Glut2 expression by HIF1α. (**a-b**) T cells were incubated overnight at 20% or 5% oxygen. Expression of Hif1α was measured by deconvolution microscopy (**a**) or flow cytometry (**b**). Representative images and histograms, and mean corrected total cell fluorescence (CTCF, **a**) or MFI (**b**) in a representative experiment are shown (±SD; n = 4-5 N = 2). (**c**) CD8 + T cells were purified from HIF+ or HIF- mice and Hif1α mRNA expression was measured by RT-PCR. Hif1α gene expression was normalized to housekeeping gene tubulin (±SD; n = 4 N = 3). (**d-e**) Glut2 (**d**) and Gal-9 (**e**) expression by 3-day antibody-activated purified naïve CD4+ T cells from Hif1+ or HIF- mice were assessed for surface expression of Glut2 by flow cytometry. Representative histograms and mean data in a representative experiment (±SD; n = 10, N = 3) (**f**) Glut2 expression by 3-day antibody-activated naïve Gal-9+ or Gal-9- CD4+ T cells was assessed by flow cytometry. Histograms and mean data from a representative experiment are shown (±SD; n = 8, N = 3). (**g**) Naïve CD4+ T cells were antibody-activated for 3 days in the presence or absence of recombinant Gal-9 (30 nM) with or without the Gal-9 competitive inhibitor lactose (30 mM) before analysing surface expression of Glut2 by flow cytometry. Representative histograms and mean data from a representative experiment are shown (±SD; n = 3, N = 2). (**h**) Expression of Gal-9 by 3-day antibody-activated CD4+ and CD8 + T cells was analysed by flow cytometry. The mean data of a representative experiment performed in triplicate is shown (±SD; n = 10 N = 3). **a-b**, unpaired, two-tailed Student’s t-test; **c**, Kruskal-Wallis test; **g**, One-way ANOVA. Source data

The observation that oxygen availability modulates Glut2 expression by T cells is consistent with a potential role for the oxygen sensor hypoxia-inducible-factor (HIF)-1α. HIF-1α is stabilized and translocates to the T cell nucleus at 5% oxygen concentration used in the experiments above (Extended Data Fig. 4a,b). Glut1 is induced by HIF-1α^26^, while HIF-1α activation has been reported to reduce Glut2 expression in muscle^27^. We therefore investigated the effect of HIF-1α pharmacological and genetic inactivation on Glut2 and Glut1 expression. Glut2 expression by CD8^+^ T cells was significantly enhanced by exposure to a selective HIF-1α inhibitor, PX-478 (ref. ^28^), both in vitro (Fig. 5a) and in vivo (Fig. 5c). As expected, expression of Glut1 was reduced by exposure to the inhibitor (Fig. 5b).

We then analysed Glut2 and Glut1 expression by T cells from *CD4*^Cre^*Hif1a*^fl/fl^ (HIF^−^) mice, in which CD8^+^ T cells also lack HIF-1α gene expression (Extended Data Fig. 4c) due to ‘leakage’ during thymic development. As shown in Fig. 5d, antibody-activated HIF^−^ CD8^+^ T cells displayed increased expression of Glut2 compared with those from *Hif1a*^fl/fl^ (HIF^+^) control mice.

Surprisingly, while Glut1 transcription was decreased in HIF^−^ CD8^+^ T cells (Fig. 5e), transcription of Glut2 was not affected by HIF-1α deficiency (Fig. 5f), suggesting that post-transcriptional mechanisms are responsible for HIF-1α-mediated modulation of Glut2 expression.

Glut2 is an *N*-glycosylated glycoprotein, whose membrane localization in pancreatic beta cells is maintained by binding to the lectin galectin-9 (Gal-9) via its *N*-glycan branches^29^. Gal-9 is a glycan-binding protein known to regulate the effector and regulatory phases of the immune response^30–32^.

HIF-1α activation upregulates Gal-9 expression^33^. Accordingly, Gal-9 expression was significantly reduced in HIF^−^ CD8^+^ T cells (Fig. 5g,h). Consistently, Gal-9 expression was increased in CD8^+^ T cells activated in low oxygen concentrations (Fig. 5i). Glut2 expression by antibody-activated Gal-9-deficient (Gal-9^−^) CD8^+^ T cells was significantly higher compared to that of their Gal-9^+^ counterparts (Fig. 5j). Vice versa, addition of recombinant Gal-9 decreased Glut2 expression by activated WT CD8^+^ T cells, and this was reversed by addition of the Gal-9 competitive inhibitor lactose (Fig. 5k)^34^.

A possible role for the HIF–Gal-9 axis in the regulation of Glut2 expression by activated CD4^+^ T cells was then investigated. Glut2 and Gal-9 expression levels were minimally increased in activated HIF^−^ CD4^+^ T cells (Extended Data Fig. 4d,e). In addition, activated Gal-9^−^CD4^+^ T cells did not display increased Glut2 expression (Extended Data Fig. 4f). Addition of recombinant Gal-9 to antibody-activated CD4^+^ T cells led to decreased Glut2 expression (Extended Data Fig. 4g), raising the possibility that CD4^+^ T cells might not produce levels of Gal-9 sufficient to downregulate Glut2 expression. Consistently, we observed that surface expression of Gal-9 is significantly lower in CD4^+^ T cells compared to CD8^+^ T cells (Extended Data Fig. 4h).

We further measured the metabolic features of Gal-9^−^ CD8^+^ T cells (Fig. 5l,m), which, as expected, displayed significantly enhanced glycolysis with no changes in the OxPhos rate.

Finally, we generated Gal-9^+^ and Gal-9^−^ BM chimeras, which received skin grafts from MHC class I mismatched B6.Kd donors. Graft rejection by Gal-9^−^ recipients was accelerated compared with that by Gal-9^+^ chimeras (Fig. 5n). However, antibody depletion of CD8^+^ T cells led to similar graft rejection kinetics by both recipients (Fig. 5o).

### Stomatin stabilizes surface expression of Glut2

While binding to Gal-9 maintains Glut2 location in non-lipid raft domains^35^, in beta cells, stomatin—a lipid raft-residing protein—directly binds to the cytosolic domain of Glut2, guiding its relocation to lipid rafts^35^.

In T cells, upregulation of stomatin expression and its location at the immunological synapse during activation increases effector responses, while its downregulation leads to loss of TCR signalling and decreased T cell activation^29^. We therefore investigated a putative role of stomatin in stabilizing the surface expression of Glut2.

**Fig. 6:**
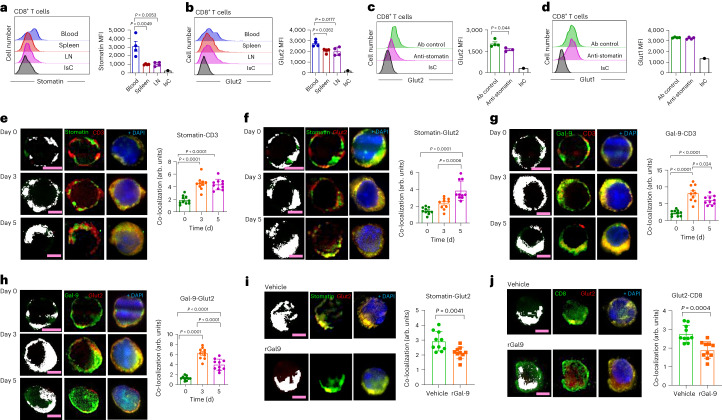
Stomatin and galectin-9 mediate regulation of Glut2 expression. **a**,**b**, Surface expression of stomatin (**a**) and Glut2 (**b**) by mouse CD44^hi^ CD8^+^ T cells from blood, spleen and LNs was analysed for by flow cytometry. Histograms and mean data (±s.d.) from a representative experiment (*n* = 3, *N* = 2). **c**,**d**, Mouse naive CD8^+^ T cells were activated by antibodies in the presence of anti-stomatin or isotype control antibody before analysing Glut2 (**c**) and Glut1 (**d**) expression by flow cytometry. Histograms and mean data (±s.d.) from a representative experiment (*n* = 4, *N* = 2). **e**–**h**, Mouse naive CD8^+^ T cells were activated by antibodies for 3 d or 5 d before analysing surface expression of Glut2, stomatin, Gal-9 and CD3 by deconvolution microscopy. Representative deconvolution and channel co-localization images of stomatin/CD3 (**e**), stomatin/Glut2 (**f**), Gal-9/CD3 (**g**) and Gal-9/Glut2 (**h**) of non-permeabilized cells at day 0 (naive) and at day 3 and day 5 after activation are shown. Co-localization of different channels is indicated by the white areas in the cell images and presented as the percentage of cell volume. Bar charts show the mean percentage of cell volume (±s.d.) in at least *n* = 10 cells from a representative of *N* = 3 experiments. **i**,**j**, Mouse naive T cells were activated by antibodies in the presence or absence of recombinant Gal-9 (30 nM) before analysing surface expression of Glut2, stomatin and CD8^+^ by deconvolution microscopy. Representative deconvolution and channel co-localization images of stomatin/Glut2 (**i**) and Glut2/CD8 (**j**) are shown. Co-localization of different channels is indicated by the white areas in the cell images and presented as the percentage of cell volume. Bar charts show the mean percentage of cell volume (±s.d.) in at least *n* = 10 cells from a representative of *N* = 3 experiments. **a**,**b**,**e**–**h**, one-way ANOVA; **c**,**d**,**i**,**j**, unpaired two-tailed Student’s *t*-test. Source data

**Extended Data Fig. 5:**
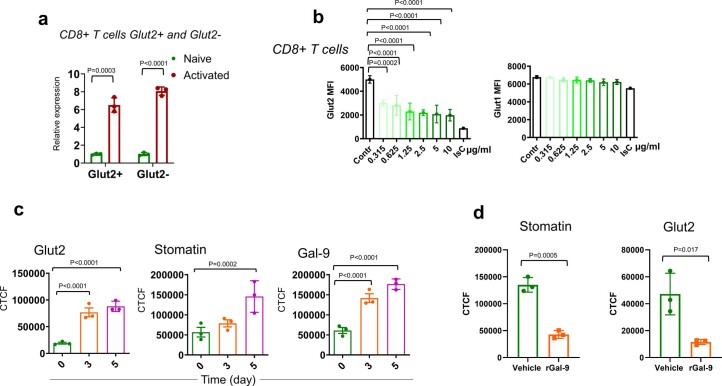
Regulation of Glut2 expression by Stomatin. (**a**) Expression of Stomatin mRNA by naïve and 3-day antibody-activated CD8 + T cells from Glut2+ or Glut2- mice was measured by RT-PCR. Stomatin gene expression was normalized to housekeeping gene tubulin (±SD; n = 3, N = 3). (**b**) T cells were antibody-activated in the presence of isotype control or anti-stomatin antibody at different concentrations before analysing Glut2 expression by flow cytometry. Mean data from a representative experiment are shown (±SD; n = 5, N = 2). (**c**) Purified CD8 + T cells were activated with plate-bound anti-CD3 (1 µg/ml) and anti-CD28 (5 µg/ml) monoclonal antibodies for 3 or 5 days before analysing surface expression of Glut2, stomatin and Gal-9 by decovolution microscopy imaging. (**d**) In some experiments, recombinant hGal-9 (30 nM) or vehicle control was added for 24 hours before staining. Bar charts show the corrected total cell fluorescence (CTCF) taken at the indicated time points. (±SD; **c**: n = 6, N = 2; **d**: n = 3, N = 2). **a**, Kruskal-Wallis test; **b-c**, One-way ANOVA; **d**, Unpaired, two-tailed Student’s T-test. Source data

Stomatin was upregulated following antibody activation (Extended Data Fig. 5a) and is expressed on the surface of CD44^hi^ T cells in secondary lymphoid organs and in blood, in which it is significantly higher, and its expression is directly proportional to that of Glut2 (Fig. 6a,b). Moreover, Glut2 but not Glut1 upregulation following antibody activation was significantly blunted by a stomatin-blocking antibody in CD8^+^ T cells (Extended Data Fig. 5b and Fig. 6c,d).

To understand the mechanistic relationship between Glut2, Gal-9 and stomatin in CD8^+^ T cells, we subsequently monitored co-localization of Gal-9, stomatin, CD3 and Glut2 by CD8^+^ T cells before (day 0) and after (day 3 and day 5) antibody activation as previously described^36^. The expression of stomatin, Gal-9 and Glut2, measured as corrected total cell fluorescence (CTCF), is shown in Extended Data Fig. 5c. As expected, stomatin co-localized with CD3 by 3 d after activation and remained stable until day 5 (Fig. 6e). In parallel, stomatin co-localization with Glut2 was still increasing 5 d after activation (Fig. 6f). In contrast, Gal-9 co-localization with CD3 was maximal 3 d after activation but declined by day 5 (Fig. 6g). Accordingly, Glut2 co-localization with Gal-9 peaked at day 3 and was significantly reduced at day 5 (Fig. 6h).

Second, we obtained evidence of a putative competition mechanism between stomatin and Gal-9 by showing that addition of exogenous recombinant Gal-9 reduced both expression (Extended Data Fig. 5d) and co-localization of both Glut2 and stomatin with CD8 (surrogate for lipid raft segregation; Fig. 6i,j). Overall, these data suggest that membrane redistribution and expression of Glut2 are dynamically orchestrated by interaction with Gal-9 and stomatin.

### Kinetics of Glut2 expression define its function in vivo

**Fig. 7:**
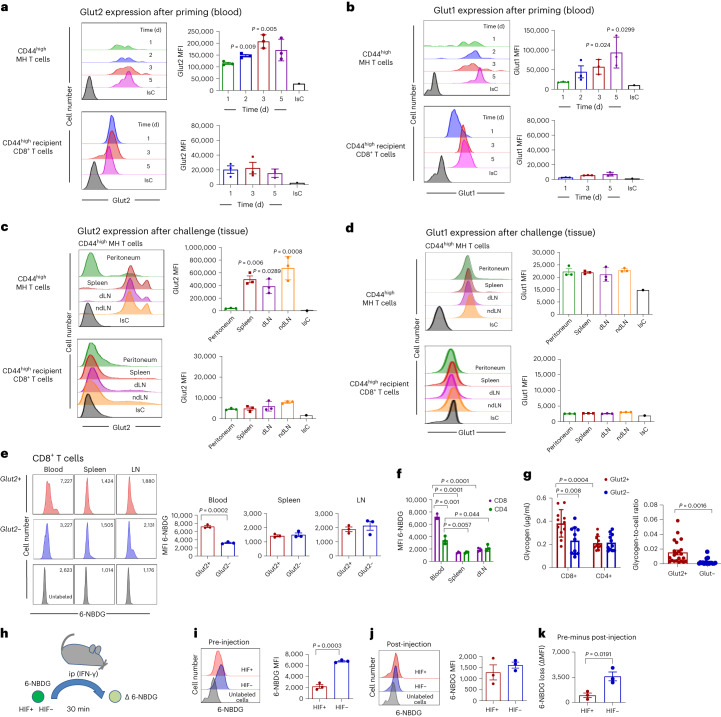
Glut2 expression and function in T cells in vivo. **a**–**d**, Purified CD45.2^+^ CD8^+^ naive T cells from MH mice were IV transferred (10^7^) into CD45.1^+^ recipients, some of which received male splenocytes (2 × 10^7^) IP a day later. Tail blood was sampled on the indicated days, and Glut2 (**a**) and Glut1 (**b**) expression by T cells were analysed. Recipient CD45.1^+^ CD44^hi^ T cells are shown for comparison. After 7 d, mice received male splenocytes (2 × 10^7^) and 1.2 µg CXCL10 IP. T cells were harvested from peritoneum, spleen, dLNs and ndLNs 16 h later. Expression of Glut2 (**c**) and Glut1 (**d**) by CD44^hi^ MH T cells and CD44^hi^ recipient CD8^+^ T cells was assessed by flow cytometry. Representative histograms are shown. The bar graphs show the MFI measured for Glut2 and Glut1 (±s.d.) in a representative experiment (*n* = 3, *N* = 2). **e**,**f**, Glut2^+^ or Glut2^−^ mice were starved for 2 h before IV injection of 6-NBDG (5 mg per kilogram body weight). Thirty minutes later, T cells were harvested from the indicated organs and 6-NBDG uptake was assessed. Representative histograms are shown. The bar graphs (**e**) show 6-NBDG MFI (±s.d.; *n* = 3, *N* = 2). **f**, Comparison of 6-NBDG uptake by CD8^+^ and CD4^+^ T cells from different tissues (±s.d.; *n* = 3, *N* = 2). **g**, Glycogen content in 3-d activated mouse Glut2^+^ and Glut2^−^ naive T cells was measured as described in the Methods. Data were normalized by protein content (mean ± s.d.; *n* = 11, *N* = 1). Right, glycogen-to-cell ratios measured by transmission electron microscope (±s.d.; *n* = 22, *N* = 1). **h**–**j**, Mouse CD45.2^+^HIF-1^+^ or HIF-1^−^ naive CD8^+^ T cells were activated by antibodies for 3 d, labelled with 6-NBDG and injected IP (10^7^) in CD45.1^+^ mice that had received IFN-γ IP 48 h previously to induce inflammation (**h**). 6-NBDG uptake by HIF^+^ and HIF^−^ T cells measured before injection (**i**) and in T cells retrieved from the peritoneal lavage 30 min after injection (**j**). Representative histograms are shown. The bar graphs show the MFI ± s.d. (*n* = 3, *N* = 3). **k**, Loss of incorporated 6-NBDG was calculated by subtracting the 6-NBDG MFI after injection to the pre-injection values (±s.d.; *n* = 3, *N* = 2). **a**–**f**, one-way ANOVA; **g**–**k**, unpaired two-tailed Student’s *t*-test. Source data

We next sought to validate the physiological relevance of Glut2 regulation by microenvironment sensing. To this aim, we used mouse CD8^+^HY-specific TCR-transgenic MH T cells. Naive CD45.2 MH T cells were adoptively transferred into CD45.1 syngeneic female recipients. The next day some mice were immunized IP with male splenocytes, and the expression of Glut2 by recirculating CD45.2^+^ CD44^hi^ (primed) CD8^+^MH T cells was assessed for the following 5 d. Glut2 expression increased on recirculating primed MH T cells, peaking 3–4 d after immunization, while it did not change on recipient memory T cells (Fig. 7a). Glut1 expression was also upregulated by primed T cells and was still increasing 5 d after immunization (Fig. 7b), as we observed following activation in vitro (Extended Data Fig. 1e,f).

**Extended Data Fig. 6:**
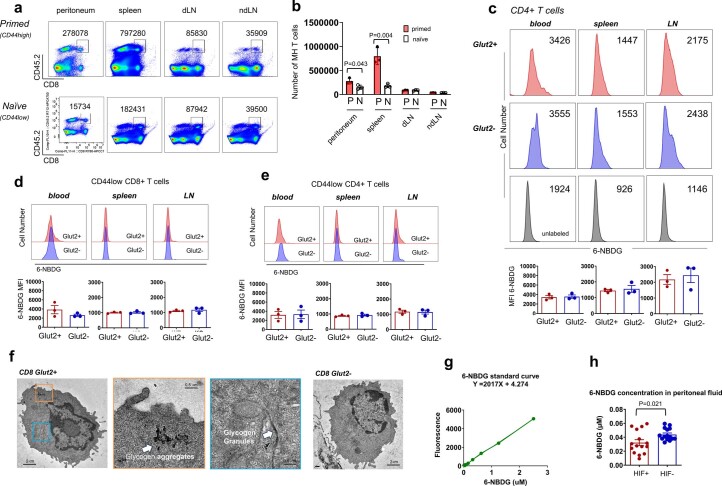
Physiologic expression and function Glut2 in CD8+ T cells. (**a-b**) Purified CD45.2+ Mata Hari CD8+ naïve T cells (MH) were adoptively transferred (10^7^) into CD45.1+ recipients (N, naïve), some of which (P, primed) received male splenocytes IP a day later. After 7 days, P and N mice received antigenic male splenocytes (2 × 10^7^) and 1.2 µg of the chemokine CXCL10 IP. T cells were also separately harvested from peritoneum, spleen, mesenteric LN (draining, dLN), and axillary LN (non-draining, ndLN) 16 hours later. The presence of primed CD44hi (P) and naïve CD44low (N) MH T cells CD8 + T cells was assessed by flow cytometry. Representative dot-plots and the mean percentage of MH T cell subset retrieved from the indicated sites in a representative experiment are shown (±SD; n = 3, N = 2). (**c**) Glut2+ or Glut2- mice were starved for 2 hours before IV injection of 6-NBDG (5 mg/kg). 30 minutes later, CD4+ T cells were harvested from blood, spleen, and LN and 6-NBDG uptake assessed. Representative histograms are shown. The bar graphs show the mean fluorescence intensity (MFI) of 6-NBDG (±SD; n = 3, N = 2). (**d-e**) 6-NBDG uptake in naïve CD44low CD8+ (**d**) and CD4+ (**e**) T cells from different tissues in the experiments described above (±SD; n = 3, N = 2). (**f**) Representative transmission electron microscopy images (magnification 3800X) of glycogen content in 3-day activated CD8+ Glut2+ and Glut2- naive T cells. White arrows point to glycogen deposits. Scale bar is indicated in each picture. (N = 2) (**g-h**) Antibody-activated (3 days) CD45.2 + HIF1+ or HIF1- CD8 + T cells were labelled with 6-NBDG and injected IP in CD45.1+ mice which had received IFN-γ ip 48 hours previously. (**g**) Standard curve of fluorescence measurement for various known 6-NBDG concentrations, from which the formula Y = 2017X + 4.274 is calculated. ‘Y’ is florescence reading and ‘X’ is 6-NBDG concentration (μM). (**h**) Mean concentrations of 6-NBDG in the peritoneal fluid (±SD; n = 6, N = 2). **a-b**, One-way ANOVA; **h**, Unpaired, two-tailed Student’s T-test. Source data

To model a non-lymphoid antigenic site, mice received a further IP injection of male splenocytes plus CXCL10 a week after priming. Glut2 expression by primed MH T cells was assessed after 16 h to avoid the confounding effect of antigen-induced T cell division. Primed MH T cells migrated to the peritoneum and spleen, indicating their recirculation out of the lymph nodes and to antigen-rich tissue. No difference was found in the distribution of naive MH T cells (Extended Data Fig. 6a,b). While maintained in secondary lymphoid organs, Glut2 expression was dramatically reduced in primed T cells migrated to the peritoneum (Fig. 7c). Glut1 expression did not significantly change (Fig. 7d).

We further investigated the physiological role of Glut2-mediated glucose uptake by T cells in different tissue environments in vivo. Addressing this question using the model above was unfeasible as 6-NBDG-labelled T cells become undetectable within 60 min from injection. Glut2^−^ or Glut2^+^ chimeras were starved for 2 h before systemic administration of 6-NBDG. Thirty minutes later, 6-NBDG uptake by T cells from blood, LNs and spleen was assessed. As shown in Fig. 7e, 6-NBDG uptake by Glut2^−^ CD44^hi^ CD8^+^ T cells was severely impaired in blood but unchanged in spleen and lymph nodes. Of note, 6-NBDG uptake in the blood was significantly higher in CD8^+^ compared with CD4^+^ Glut2^+^ T cells, while uptake in lymph nodes and spleen was similar in both subsets (Fig. 7f). No difference in glucose uptake was observed in Glut2^+^ and Glut2^−^ naive (CD44^lo^) CD8^+^ and CD4^+^ T cells (Extended Data Fig. 6d,e). Thus, compared to memory CD4^+^ T cells, primed CD8^+^ T cells physiologically take up a large amount of glucose while recirculating in the blood in a Glut2-dependent manner.

In CD8^+^ lymphocytes, glucose taken up is partially stored as glycogen^37^. As shown in Fig. 7g and Extended Data Fig. 6f, intracellular glycogen is significantly higher in Glut2^+^ memory CD8^+^ T cells compared to CD4^+^ T cells and glycogen stores are depleted in Glut2^−^ CD8^+^ but not CD4^+^ T cells.

On this basis, we took advantage of the reduced ability of HIF^−^ CD8^+^ T cells to downregulate Glut2 to challenge the hypothesis that the substantial downregulation of Glut2 expression by CD8^+^ T cells in inflammatory sites might prevent glucose leakage. Equal numbers of HIF^+^ and HIF^−^ naive CD8^+^ T cells were activated by antibodies for 3 d, labelled with 6-NBDG and injected IP in mice that had received IFN-γ and CXCL10 IP 48 h earlier to generate an inflammatory site (Fig. 7h). Uptake of 6-NBDG was measured before and 30 min after injection. As expected, glucose uptake by CD8^+^HIF^−^ T cells was significantly higher than that of HIF^+^CD8^+^ T cells (Fig. 7i). However, when 6-NBDG expression was measured after retrieving the T cells, 6-NBDG content was similar in HIF-1^+^ and HIF-1^−^ CD8^+^ T cells (Fig. 7j). Differential glucose leak was calculated by subtracting 6-NBDG mean fluorescence intensity (MFI) in T cells retrieved in the peritoneum from that displayed before injection and showed a significantly higher loss of 6-NBDG by HIF^−^ T cells (Fig. 7k). A comparison of the peritoneal fluids showed a significantly higher 6-NBDG concentration in recipients of HIF^−^ CD8^+^ T cells (Extended Data Fig. 6g,h).

### Glut2 regulates human T cell responses

Inactivating mutations of GLUT2 in humans cause Fanconi–Bickel syndrome, a severe paediatric condition associated with recurrent infections particularly of the lower respiratory tract^38,39^.

To assess a potential role of GLUT2 in the human system, we analysed T cells from carriers of a rare polymorphism in the GLUT2-encoding *SLC2A2* gene (single-nucleotide polymorphism (SNP) rs5400 (p.Thr110Ile); Supplementary Table 1). Albeit not severely affecting glucose uptake by Glut2, p.Thr110Ile is associated with decreased fasting plasma glucose and insulin^40^.

**Fig. 8:**
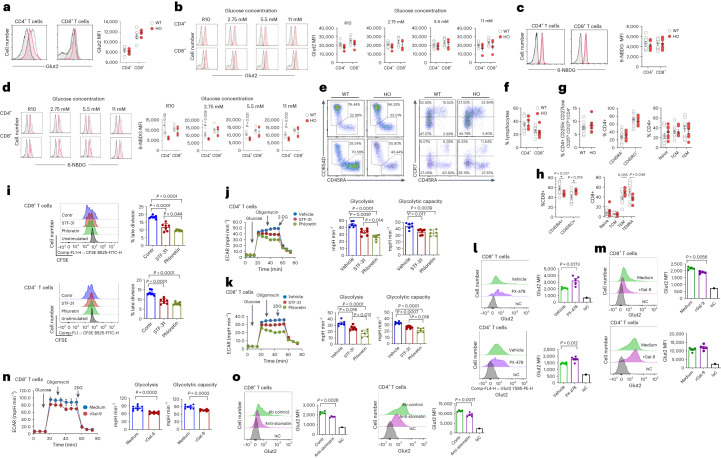
Glut2 is expressed by, and is functional in, human T cells. **a**–**d**, GLUT2 expression (**a** and **b**) and 6-NBDG uptake (**c** and **d**) by human CD4^+^ and CD8^+^ T cells from WT and age-matched and sex-matched homozygous carriers of SLC2A2 SNP (HO) ex vivo (**a** and **c**) or after 2 d of antibody activation in complete RPMI (R1) or glucose-free medium reconstituted with the indicated concentrations of glucose (**b** and **d**); representative histograms (grey line indicates controls, red line indicates SLC2A2 SNP carriers, black indicates FMO control staining) and mean data measured in the indicated number of individuals ± s.e.m., *n* = 4–10 per group, *N* = 1. **e**–**h**, Representative dot plots and percentage of circulating human CD4^+^ and CD8^+^ T cells (**e**) and their subsets (**f** and **g**, respectively) in WT and HO individuals: regulatory T cells (CD4^+^CD25^hi^CD127^lo^), naive (CD45RA^+^CCR7^+^), central memory (T_CM_, CD45RA^−^CCR7^+^), effector memory (T_EM_, CD45RA^−^CCR7^−^) and terminally differentiated effector memory T cells (T_EMRA_, CD45RA^+^CCR7^−^; ±s.d.; *n* = 7 per group, *N* = 1). **i**, CFSE (5 µM)-labelled human CD8^+^ and CD4^+^ T cells were activated by antibodies for 4 d in the presence of Glut1 inhibitor (STF-31, 1.25 µM) or dual inhibitor (phloretin, 75 µM) or vehicle added for the last 24 h. Representative histograms and mean data representative of three independent experiments performed in triplicate are shown (±s.d.; *n* = 7, *N* = 1). **j**, ECAR, mean glycolysis and glycolytic capacity in 4-d activated human CD4^+^ T cells treated with Glut1 inhibitor (STF-31, 1.25 µM) or dual inhibitor (phloretin, 75 µM) or vehicle control. (±s.d., *n* = 6, *N* = 1). **k**, ECAR, mean glycolysis and glycolytic capacity in 4-d activated human CD8^+^ T cells treated with Glut1 inhibitor (STF-31, 1.25 µM) or dual inhibitor (phloretin, 75 µM) or vehicle control. (±s.d., *n* = 6, *N* = 1). **l**, Glut2 expression by antibody-activated human naive T cells (6 d) in the presence or absence of the HIF-1α selective inhibitor PX-478 (20 µM) (±s.d.; *n* = 4–5, *N* = 1). **m**, Glut2 expression by antibody-activated human naive T cells (6 d) in the presence or absence of recombinant Gal-9 (30 nM) (±s.d.; *n* = 4–5, *N* = 1). **n**, ECAR, mean glycolysis and glycolytic capacity in 4-d activated human CD8^+^ T cells treated with recombinant Gal-9 (30 nM) or vehicle control. (±s.d., *n* = 8, *N* = 1). **o**, Human naive CD3^+^ T cells were activated by antibodies for 6 d in the presence of anti-stomatin (2.5 μg ml^−1^) or isotype control antibody before analysing surface expression of Glut2 (*n* = 4–5, *N* = 1). **a**–**h**,**k**–**o**, unpaired two-tailed Student’s *t*-test; **i**–**k**, one-way ANOVA. Source data

CD4^+^ and CD8^+^ T cells were isolated from peripheral blood mononuclear cells (PBMCs) and expression of Glut2 was assessed by flow cytometry. As shown in Fig. 8a, ex vivo expression of Glut2 in CD4^+^ and CD8^+^T cells was similar in WT and p.Thr110Ile SNP carriers, irrespective of the amount of glucose in the cultures (Fig. 8b). However, 6-NBDG uptake, despite similar expression levels after ex vivo incubation (Fig. 8c), was significantly reduced in both p.Thr110Ile CD4^+^ and CD8^+^ T cells following antibody activation, irrespective of the amount of glucose (Fig. 8d), indicating that Glut2 contributes to glucose uptake in human T cells but, unlike in mouse T cells, glucose concentrations do not affect its expression.

We further observed a significant decrease in the proportion of T_EMRA_ (CD45RA^+^, CCR7^−^) CD8^+^ T cells in p.Thr110Ile SNP carriers, which was accompanied by an increase of T cells with an effector-memory phenotype (Fig. 8e–h), in line with the observation made in Glut2^−^ murine T cells, which displayed reduced terminal differentiation both in vitro and in vivo (Extended Data Fig. 2c,d), although T_EMRA_ cells are not detectable in mice^41^.

**Extended Data Fig. 7:**
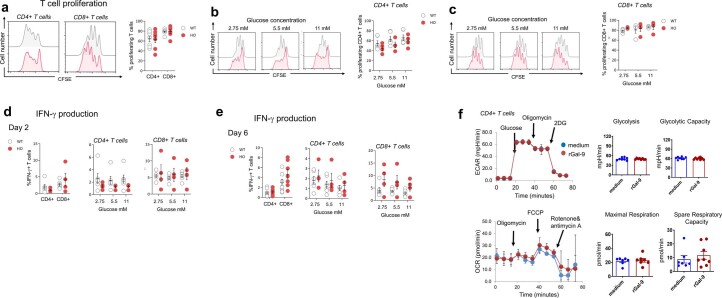
Function of Glut2 in human T cells. (**a-c**) Mean percentage of proliferating CD4+ and CD8 + T cells from wild type (WT) and matched age and sex homozygous carriers of SLC2A2 SNP (HO) after 6 days of antibody activation in complete RPMI medium (**a**), or in glucose-free medium reconstituted with 2.75 mM, 5.5 mM or 11 mM glucose (**b-c**) ( ± SEM; n = 4-9, N = 1). (**d-e**) Percentage of IFN-γ+ total, CD4+ and CD8 + T cells from wild type and matched age and sex carriers of SLC2A2 SNP after 2 or 6 days of activation in complete RPMI medium or glucose-free DMEM reconstituted with 2.75 mM, 5.5 mM or 11 mM glucose, 2 and 6 days after antibody activation ( ± SEM; n = 4-9, N = 1). (**f**) Extracellular acidification rate (ECAR, mpH/min) and oxygen consumption rate (OCR; pmol/min) in 4-day activated human CD4+ T cells treated with recombinant Gal-9 (30 nM) or vehicle control. The bar graph shows the mean glycolysis, glycolytic capacity, maximal respiration, and spare respiratory capacity ( ± SEM, n = 7-8, N = 1). Unpaired, two-tailed Student’s T-test. Source data

We did not detect differences between WT and p.Thr110Ile carriers in terms of T cell proliferation and IFN-γ production (Extended Data Fig. 7a–e).

Given the limitations imposed by the relatively small number of p.Thr110Ile SNP carriers and the mild effect of this SNP on Glut2 function, we investigated the effects of pharmacological GLUT2 and GLUT1 inhibition in human T cells purified from blood buffy coats. To this aim, we used the dual inhibitor phloretin and the GLUT1-selective inhibitor STF-31 (ref. ^16^).

Proliferation of CD4^+^ and CD8^+^ T cells was reduced by both inhibitors (Fig. 8i), although this effect was greater in CD8^+^ T cells, suggesting that GLUT2 is a main contributor to CD8^+^ human T cell proliferation. We subsequently measured ECAR of 4-d activated CD4^+^ and CD8^+^ T cells. Inhibition of GLUT1 and GLUT2 led to a significant decrease of glycolysis in both T cell subsets, although this was more profound when T cells were exposed to phloretin, indicating a contribution of both transporters to the glycolytic pathway (Fig. 8j,k). Of note, exposure to phloretin did not reduce the glycolytic flux to the extent observed in mouse T cells (Extended Data Fig. 1i,j), raising the possibility that additional glucose transporters might be operational in human T cells.

Like in mouse T cells, pharmacological inhibition of HIF-1α led to upregulated expression of Glut2 in CD8^+^ T cells (Fig. 8l). Exposure of human T cells to Gal-9 decreased GLUT2 expression (Fig. 8m) and glycolysis in CD8^+^ (Fig. 8n) but not CD4^+^ (Extended Data Fig. 7f) T cells. Finally, antibody-inhibition of stomatin led to a decrease of Glut2 expression in both CD4^+^ and CD8^+^ T cells (Fig. 8o).

Overall, these data suggest that Glut2 plays a role in human T cell glucose metabolism and, like in mouse T cells, is regulated by the interplay of HIF-1α, Gal-9 and stomatin.

## Discussion

We here report a dominant contribution of the glucose transporter Glut2 in the effector phase of CD8^+^ T cell-mediated immune responses.

Glut2 promotes CD8^+^ T cell proliferation, differentiation and function by fuelling glycolysis during activation and is instrumental to glucose uptake and glycogen synthesis during CD8^+^ T cell recirculation after priming, required for optimal CD8^+^ T cell function at effector sites^37^. In line with our findings, efficient glycolysis is required to promote IFN-γ gene expression^18,19^, including in CD8^+^ T cells^42^. Also, glucose-deficient CD8^+^ T cells have impaired cytolytic activity, including reduced granzyme and perforin production^43^. Finally, our data are consistent with reports that attenuation of glycolysis is associated with the development of central memory T cells^44,45^.

The dynamic regulation of Glut2 expression at different phases and sites suggests a unique role of this glucose transporter in facilitating metabolic adaptation of CD8^+^ T cells in distinct microenvironments, likely defined by its low affinity for glucose. First, Glut2 is highly expressed by recently primed T cells in the blood, which is rich in glucose and highly oxygenated. High glucose uptake and glycogen storage during recirculation are essential to CD8^+^ T cell survival in inflammatory sites of effector response, as previously reported^37^.

In contrast, Glut2 is dramatically downregulated in poorly oxygenated inflammatory/tumour sites (inflamed peritoneum and tumour). Given the ability of low-affinity Glut2 to bidirectionally transport glucose along a concentration gradient, its prompt removal from the cell surface is instrumental to prevent glucose leakage in glucose-depleted environments. In contrast, the high-affinity Glut1, whose expression does not decrease in inflammatory sites, can sustain glucose uptake in conditions of low glucose availability.

Mechanistically, the different effects of oxygen sensing mediated by the transcription factor HIF-1α dominate the differential regulation of Glut2 and Glut1 expression. As both transporters are regulated in a similar manner by glucose concentration and pH, the opposite regulatory outcomes by HIF-1α activation on Glut2 and Glut1 expression are instrumental to this complex, yet extremely effective coordinated function of the two transporters.

**Extended Data Fig. 8:**
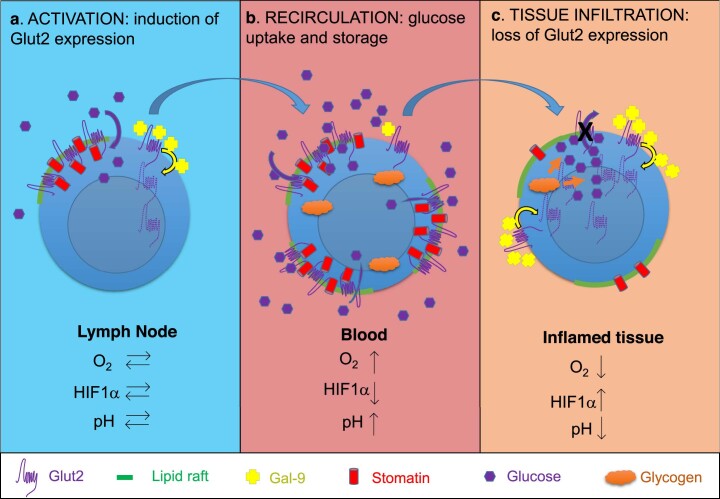
Regulation of Glut2 expression in CD8+ T cells. (**a**) Upon T cell activation expression of Glut 2 is upregulated on the T cell surface, where it localizes in lipid rafts via association with Stomatin. Stomatin competes with Galectin-9 (Gal-9) which segregates Glut2 in the non-raft membrane, where it is internalized. (**b**) Once T cells reach the circulation, they are exposed to increased glucose concentration, oxygen tension, and physiological pH which, by inhibiting HIF1α activation and subsequent Gal-9 transcription, further promote Glut2 expression, glucose uptake and storage as Glycogen. (**c**) Once in the inflammatory site, a drop in oxygen tension and pH upregulate Gal-9 expression, removal of Glut2 from lipid raft domains and subsequent internalization. This prevents passive loss of intracellular glucose in conditions of low extracellular availability.

Intriguingly, while HIF-1α directly and positively regulates Glut1 expression at the transcriptional level, inhibition of Glut2 expression is indirect and involves increased Gal-9 production and competition with stomatin for its localization in rafts at the cell membrane. Following T cell activation in the lymph nodes, stomatin is upregulated and can compete with Gal-9, thus stabilizing Glut2 at the cell membrane. In contrast, upregulation of Gal-9 by HIF-1α activation outcompetes stomatin leading to Glut2 downregulation. In this respect, regulation of Glut2 membrane stabilization follows a similar pattern to that described for pancreatic beta cells^35^, albeit with opposite effects. The molecular basis of the T cell-specific effects remains to be established, and it is likely to involve interactions of Glut2 with distinct, yet-undefined partners in the lipid raft microdomains. In this context, multivalent galectin 3–*N*-glycan complexes have been shown to reduce TCR clustering by restricting lateral TCR movement within the plane of the membrane, thereby increasing the agonist threshold for TCR signalling^46^. Similarly, Glut1 has been shown to interact with stomatin in adipocytes leading to its translocation in lipid rafts^47^. These interactions were enhanced in glucose deprivation conditions, but did not affect Glut1 ability to uptake glucose, suggesting that stomatin may also serve as an anchor for Glut1 in lipid rafts. A summary of these observations is provided in Extended Data Fig. 8.

The physiological relevance of relative lack of effect of Glut2 deficiency in CD4^+^ T cells is intriguing. Given that the effect of Gal-9 on Glut2 expression occurs in antibody-activated CD8^+^ T cells, it must be assumed that in this subset this effect is operated in an autocrine manner. In contrast, in CD4^+^ T cells, Glut2 expression is downregulated only by addition of exogenous Gal-9, suggesting that these T cells do not produce sufficient Gal-9 to elicit autocrine effects. This difference might underlie the fact that CD4^+^ T cells have adapted their metabolic responses towards a dominant Glut1 function^12^.

The relative contribution of these glucose transporters appears to be clearly demarcated by the environmental conditions in which CD4^+^ and CD8^+^ T cells develop and exert their effector functions. In the CD8^+^ T cell subset, both Glut1 and Glut2 can support the glycolytic pathway. The prominent dependence of CD8^+^ T cells on Glut2 expression is likely dictated by a combination of requirement for glucose availability and glycolysis to sustain effector responses^5^, including glycogen storage^37^ for intracellular supply, and the location of activities in severely glucose-depleted microenvironments. It is likely that the role of Glut1, as a transporter that can function in conditions of low glucose availability, plays an auxiliary role when CD8^+^ T cells localize to glucose-depleted inflammatory sites, and lose Glut2 expression. The relative Glut2 independence of CD4^+^ T cells might reflect the fact that effector CD4^+^ T cells carry out a large part of their activities such as cytokine production and help for B cell responses in lymph nodes, which provide a less harsh microenvironment compared to that experienced by effector CD8^+^ T cells.

The relative contribution of Glut1 and Glut2 in human CD4^+^ and CD8^+^ T cell responses is less clear cut. In our pilot study, both glucose transporters equally contribute to T cell glucose uptake, glycolytic activity and T cell division. However, the differential regulation of their expression appears conserved in mice and humans, suggesting that the prominent environmental dependence of the transporters is conserved. Further studies are needed to fully resolve these species-specific differences.

Finally, the present study fully supports the notion that in vitro studies of metabolism need to reproduce the environmental cues that T cells encounter in vivo, during recirculation in different tissues compartments^48^. As we have shown, glucose availability and oxygen concentration and pH are other parameters that should be considered when studying metabolic adaptation of T cells, which in physiology constitutively and continuously visit different microenvironments, in vitro.

## Methods

### Study approvals

All animal experiments were approved by Queen Mary Ethics of Research Committee in Queen Mary University of London (establishment licence number: XEDA0F7B1), and conducted with strict adherence to the Home Office guidelines (PPL P71E91C8E).

Human blood was obtained from healthy donors from the Progressione della Lesione Intimale Carotidea (PLIC) study (a sub-study of the CHECK study), a large survey of the general population of the northern area of Milan (*n* = 2,606)^49^, approved by the Scientific Committee of the Università degli Studi di Milano (ref. SEFAP/Pr.0003-Fa-04-Feb-01). Informed consent was obtained from participants in accordance with the Declaration of Helsinki. Donor characteristics are available in the Supplementary Information and Supplementary Table 1.

### Statistical analysis

Results are expressed as the mean and s.d. or the mean and s.e.m. The data met the assumptions of the statistical tests used, including whether normality and equal variances were formally tested. Unpaired student’s *t*-test, one-way ANOVA and log-rank (Mantel–Cox) test were used. Data distribution was assumed to be normal, but this was not formally tested. All reported *P* values are two sided. Throughout the text, the number of independent biological samples or animals/groups in the same experiment is indicated by *n* = *x*, and the number of independent experiments is indicated by *N* = *x*. Statistical analysis was carried out using Prism v9. Except for sex, animals/samples were randomly assigned to the various experimental groups. Single blinding was used in transplantation and tumour models. No animals or data points analysed at the end of experiments were excluded from the analyses. No statistical methods were used to predetermine sample sizes, but our sample sizes are similar to those reported in previous publications^5,23,50^.

### Animals

C57BL/6J mice were purchased from Charles River (UK). Ripglut1;*Glut2*^−/−^ mice (referred to as Glut2^−^) have been previously described^15^. B6.Kd (BL/6 transgenic for Kd) were a gift from R. Lechler (King’s College London). B6.129-Hif1atm3Rsjo/J (HIF1a^loxp^) and B6.Cg-Tg(CD4-cre)1Cwi/BfluJ mice were purchased from Jackson Laboratory. Gal-9 knockout mice (B6(FVB)-Lgals9tm1.1Cfg/Mmucd) on a C57BL/6J background were obtained from the Mutant Mouse Resource and Research Centers (originally deposited by J. Paulson, The Scripps Research Institute). Marilyn mice, bearing a transgenic TCR specific for the male minor transplantation antigen HY peptide epitope Dby (NAGFNSNRANSSRSS) and restricted by H2-Ab molecules, and MH mice, bearing a transgenic TCR specific for the male minor transplantation antigen HY peptide epitope Uty (WMHHNMDLI) and restricted by H2-Db molecules have been previously described^21,22^. Mice were fed a regular chow diet and used at the age of 8–12 weeks. Littermates of the same sex were randomly assigned to experimental groups. In experiments assessing T cell response to HY antigen, only female mice were used.

To generate chimeric mice, C57BL/6J mice were sub-lethally irradiated with two doses of 4-Gy X-ray delivered 4 h apart. Eighteen hours after the second dose of irradiation, they were reconstituted with 3 × 10^6^ BM cells harvested from age-matched WT or Ripglut1;*Glut2*^−/−^ mice (referred to as Glut2^−^) or B6(FVB)-Lgals9tm1.1Cfg/Mmucd mice (referred to as Gal-9^−^). After 8 weeks, mice were tested for chimerism by RT–PCR.

Mice were kept at 18–23 °C and 40–60% humidity with a 12-h light–dark cycle.

### Reagents

Unless otherwise indicated, all the experiments were performed in medium supplemented with 5 mM glucose. A detailed list of reagents is available in the Supplementary Information.

### Antibodies

For the phenotypic characterization of mouse studies, cells were stained with: CD25 PE (clone PC61.5); CD26 PerCP/Cy5.5 (clone H194-112); CD28 PE (clone 37.51); CD31 PE/Cy7 (clone 390); CD38 PE/Cy7 (clone 90); CD44 pacific blue or BV605 (clone IM7); CD45.1 FITC (clone A20); CD45.2 AF700 (clone 104); CD49d PE (clone 9C10); CD62L FITC (clone MEL-14); CD69 FITC (clone H1.2F3); Glut1 AF647 (clone EPR3915) or AF 405 (polyclonal); Glut2 PE (clone 205115); Gal-9 APC (clone 108A2) or PE/Cy7 (clone RG9-35); stomatin AF488 (polyclonal); MHC class I (H-2kb/H-2Db) AF647 (clone 28-8-6); TCRβ BV605 (clone H57-597); CCR4 APC (clone 2G12); CCR5 PE (clone HM-CCR5); CCR6 PE (clone 29-2L17); CCR7 APC (clone 4B12); CXCR3 PerCP/Cy5.5 (clone CXCR3-173); CXCR4 FITC (clone 2B11/CXCR4); LFA-1 PE/Cy7 (clone M17/4); B220 BV605 (clone RA3-6B2); and CD107α PE/Dazzle 594 (clone 1D4B);

Phenotypic characterization of human cells was performed with: CD3 AF700 (clone UCHT1); CD4 BV605 (clone SK3, also known as Leu3a); CD8 ef450 (clone SK1); CD8 FITC (clone RPA-T8); CCR7 FITC (clone REA546); CD45RA-BV785 (clone HI100); CD45RA-PerCPVio700 (clone REA1047); CD45RO, APCVio770 (clone REA611); and Glut2 PE (clone 199017).

Intracellular staining for mouse studies was performed with the following antibodies: FoxP3 APC (clone FJK-16s); HIF-1α FITC or PE (clone 241812); granzyme B FITC (clone GB11); IFN-γ PerCP/Cy5.5 (clone XMG1.2); and IL-17 ef450 (clone eBio17B7). IFN-γ AF647 (clone B27) was used for intracellular cytokine staining in human cells.

All antibodies were used at a 1:200 dilution for flow cytometry and 1:50 dilution for imaging.

### Cell surface and intracellular staining

A step-by-step protocol for surface or intracellular staining is provided in Supplementary Methods.

Samples were acquired on a six-laser Beckman Coulter CytoFLEX LX collected by CytExpert 2.4 (Beckman). Setup and tracking beads (BD) were routinely used to calibrate the cytometer. Single-stain controls and fluorescence-minus-one (FMO) controls were acquired for compensation and precise gating, respectively. Compensation was automatically calculated, and samples were analysed using FlowJo (v10.6.2).

### Antibody-mediated T cell activation

T cells were stimulated with plate-bound anti-CD3 (1 μg ml^−1^, eBiosciences, 16-0032-85) and anti-CD28 (5 μg ml^−1^, eBiosciences, 16-0281-86) in cell medium supplemented with 20 U ml^−1^ recombinant IL-2 (Roche, 10799068001). To evaluate the effect of oxygen, T cells were activated with antibodies for 2 d, then transferred into incubators containing either 20% oxygen or 5% oxygen (Baker Ruskinn InvivO_2_ 400 hypoxic workstation) overnight before analysis.

In experiments investigating the effect of pH on Glut2 expression, various volumes of 1 M HCl were added to the medium until the desired pH was obtained. Before use in experiments, the medium pH was checked after 24 h of incubation at 37 °C under 5% CO_2_.

In polarization studies, CD8^+^ T cells were isolated from WT and Glut2-deficient mice, activated by plate-bound CD3/CD28 antibodies, and differentiated towards the Tc-0 and Tc-1 phenotypes by culturing with 10 ng ml^−1^ IL-2 (Tc-0) or 10 ng ml^−1^ IL-2; 5 ng ml^−1^ IL-12 (BioLegend, 577002) and 2 μg ml^−1^ anti-IL-4 (BioLegend, 504102; Tc-1).

### Measurement of ECAR and OCR

Real-time bioenergetics analysis of ECAR and OCR of T cells was performed using the XF Analyzer (Seahorse Biosciences). Mouse CD8^+^ and CD4^+^ T cells were isolated and activated with antibodies for 2 d. Human CD8^+^ and CD4^+^ T cells were isolated from PBMCs and activated with antibodies for 5 d. Glut1 inhibitor (STF-31, 1.25 µM) or Glut2 inhibitor (phloretin, 75 µM) or vehicle control was added to cells 1 h before Seahorse assay. Recombinant Gal-9 (30 nM) or vehicle control was added to cells 1 d before and during the Seahorse assay. T cells were seeded (3–5 × 10^5^ per well) into the Seahorse XF96 cell plates for analysis. Perturbation profiling of the use of metabolic pathways by T cells was achieved by the addition of oligomycin (1 μM), FCCP (1 μM), antimycin A (1 μM), rotenone (1 μM), d-glucose (10 mM) and 2-deoxy-d-glucose (50 mM).

### Metabolic labelling and metabolome analysis

Naive CD8^+^ T cells were isolated from Glut2^+^ and Glut2^−^ mice and activated with antibodies in glucose-free RPMI supplemented with 2.5 mM glucose for 48 h. Cells were the cultured in medium containing 5 mM ^13^C_6_-glucose (Cambridge Isotope Laboratories, CLM-1396-5) for an additional 18 h before metabolite isolation. Briefly, cells were washed with PBS three times and resuspended in ice-cold extraction buffer (20% ultrapure water, 50% methanol, 30% acetonitrile) at a ratio of 20 × 10^6^ cells per ml. Cells were incubated on methanol and dry ice for 15 min, placed on a shaker for 15 min at 4 °C, then at –20 °C for 1 h. The cell lysate was centrifuged, and the supernatant was collected and transferred to autosampler glass vials, which were stored at –80 °C. LC–MS/MS analysis was performed using a Q Exactive Quadrupole-Orbitrap mass spectrometer coupled to a Vanquish UHPLC system (Thermo Fisher Scientific; Supplementary Methods). Features with a fold change greater than 2 and *P* < 0.05 were selected as discriminating markers. Samples were analysed in quadruplicate.

### RT–qPCR

RNA was purified using Qiagen RNAeasy Kit according to the manufacturer’s instructions. Reverse transcription was performed according to the manufacturer’s instructions (Applied Biosystems). Gene expression analysis was done using SYBR Green Supermix (Bio-Rad) in CFX-Connect RT–PCR System and CFX Manager Software version 2.1 (Bio-Rad), according to the manufacturer’s instructions. The qPCR data were analysed using the delta-delta CT method.

### 6-NBDG uptake assays

T cells were washed in PBS and resuspended in glucose-free T cell medium for 1 h before a final concentration of 60 μM 6-NBDG in glucose-free T cell medium was added to the cells. Cells were further incubated for an additional 30 min. Finally, the cells were washed twice with warm PBS and resuspended in flow cytometry buffer and placed on ice. Immediate analysis was performed using flow cytometry to observe 6-NBDG uptake by the T cells.

For in vivo uptake experiments, mice were starved for 2 h before injection of 6-NBDG at 5 mg per kilogram of body weight. Cells were harvested 30 min later, washed twice with warm PBS and resuspended in flow cytometry buffer and placed on ice. Flow cytometric analysis was performed immediately.

### In vivo proliferation

T cells from Marilyn mice (10^7^ cells per mouse) and MH mice (10^7^ cells per mouse) were labelled with CFSE (5 μM) and injected intravenously into Glut2^+^ and Glut2^−^ female recipients. Twenty-four hours later, recipient mice received an IP injection of male splenocytes (5 × 10^6^). Five days after immunization, T cells were separately harvested from mesenteric LNs (dLNs), inguinal and axillary LNs (ndLNs) and the spleen. CFSE dilution in Marilyn T cells (CD4^+^Vb6^+^CD45.2^+^) and MH T cells (CD8^+^Vb8.3^+^CD45.2^+^) was assessed by flow cytometry.

### Immunization with ovalbumin

Mice were primed and boosted with an IP injection of 750 µg ovalbumin protein plus 50 µg poly(I:C) adjuvant or adjuvant alone as control. After 7 d, T cells were separately harvested from mesenteric LNs (dLN), inguinal and axillary LNs (ndLNs) and the spleen. Expression levels of IFN-γ, granzyme B and IL-17 were assessed by flow cytometry.

### Glycogen quantification using a glycogen assay kit

Glycogen levels were measured using a glycogen assay kit (Merck) following the manufacturer’s instructions. Briefly, 1 × 10^6^ day 3-activated T cells were homogenized with 200 μl water on ice and then boiled for 10 min. Homogenates were spun at 20,000*g* for 10 min, and supernatants were assayed for glycogen content by SPECTROstar Omega using software V5.11R4. Results were normalized by protein content.

### Glycogen quantification by transmission electron microscope

Naive Glut2^+^ and Glut2^−^ CD8^+^ T cells were purified, activated with antibodies for 4 d and harvested for imaging of glycogen by transmission electron microscope. Briefly, cell samples were embedded in 2% (wt/vol) low-gelling-temperature agarose, cut in 1–2-mm cubic blocks, and fixed with 2% (wt/vol) potassium permanganate dissolved at 4 °C overnight. Samples were washed with distilled water and dehydrated through a graded ethanol series. Samples were then washed twice with propylene oxide before infiltration with Araldite for 1 h and with fresh Araldite overnight. Polymerization was achieved by incubation at 60–65 °C for 48 h. Alternatively, cells were fixed at room temperature for 2 h in 100 mM phosphate buffer pH 7.0 containing 0.5% (wt/vol) tannic acid, 1% (wt/vol) formaldehyde and 3% (wt/vol) glutaraldehyde, washed with phosphate buffer, and incubated in 2% (wt/vol) OsO_4_ in phosphate buffer overnight. Dehydration was performed using a graded acetone series. Thin sections were cut with a glass knife at a Reichert Ultracut E microtome and collected onto uncoated 300-mesh copper grids. High contrast was obtained by post-staining with saturated aqueous uranyl acetate and lead citrate for 4 min each. The grids were examined in a JOEL JEM-1230 transmission electron microscope. Areas of glycogen deposits and whole cells were quantified and analysed via QuPath V0.43 and GraphPad 9.

### In vivo glucose leakage in inflamed non-lymphoid tissue

Purified CD45.2^+^HIF-1α-competent or HIF-1α-deficient naive CD8^+^ T cells (10^7^) were activated with antibodies for 3 d, labelled with 6-NBDG and injected IP in CD45.1^+^ syngeneic mice that had received an IP injection of IFN-γ (300 ng) 48 h previously. Then, 6-NBDG in CD8^+^ T cells was measured by flow cytometry before and 30 min after injection. The concentration of 6-NBDG in peritoneal fluids was measured by a fluorescence plate reader 30 min after injection.

### Skin grafting

Sections of donor tail skin (1 cm^2^) were placed onto a graft bed created on the right flank of recipient mice anaesthetized using isoflurane (Halocarbon Products). The graft was covered with muslin, and a plaster cast was wrapped around the midriff and graft. Seven to nine days later, plasters were removed. Skin graft rejection was assessed as previously described^50^.

### E0771 tumour cell implantation

For orthotopic implantation as a syngeneic mouse model of breast cancer, E0771 mouse mammary adenocarcinoma cells (4 × 10^5^) (ABC-TC5564, Accegen Biotechnology) were resuspended in a 1:1 ratio of PBS and growth-factor-reduced Matrigel (Corning; 50 µl total volume) and injected into the right fourth inguinal mammary gland of female Glut2^+^ or Glut2^−^ chimera mice.

Tumour growth was monitored every 2–3 d for 28 d by measuring tumour length and width using callipers. After harvest, tumour volume (mm^3^) was measured using the formula: [(width)2 × length]/2. Pieces of tissue were embedded in either OCT or formalin for histological examination, while other pieces of tissue were digested enzymatically using collagenase to generate single-cell suspensions for flow cytometry staining.

### Nuclei isolation and staining

T cells were incubated at 37 °C in incubators containing either 20% oxygen or 5% oxygen for 24 h before isolation of nuclei^51^. Cells were then pelleted by centrifugation, resuspended in the cell lysis buffer (10 mM HEPES; pH 7.5, 10 mM KCl, 0.5 mM EDTA, 1% Triton-X 100, to which 1 mM dithiothreitol and 0.5 mM phenylmethylsulfonyl fluoride were added just before use), placed on ice for 15–20 min with intermittent mixing by vortexing to disrupt T cell membranes. Nuclei were washed twice with the cell lysis buffer and subsequently resuspended in Fix/Perm buffer for at least 30 min before antibody staining.

### Imaging

Naive T cells were allowed to adhere onto coverslips for 30 min at room temperature. Activated T cells for staining were obtained by seeding naive T cells (1.0 × 10^6^ cells per well) onto coverslips pre-coated with anti-CD3 (1 μg ml^−1^) and anti-CD28 (5 μg ml^−1^) in 24-well plates and cultured for 3 d or 5 d. Cells were spun down with 200*g* for 1 min, followed by fixing with 2% PFA for 20 min at room temperature. Cells were then washed with PBS and blocked with blocking buffer (1% BSA, 5% goat serum) for 2 h at room temperature. After blocking, the cells were labelled with appropriate antibodies in the same blocking buffer for 48 h at +4 °C in the dark. After multiple PBS washes, coverslips were mounted onto slides with Fluoroshield and then examined using a Zeiss Z1 deconvolution microscope (Carl Zeiss) equipped with an AxioCam MRm cooled monochrome digital camera and an ApoTome.2 imaging unit. Some images were examined using a Leica SP5 confocal microscope. Confocal images and *z* stacks were acquired and analysed by Leica LAS software. Images were acquired using a ×63 1.4 NA (oil) objective.

The CTCF was measured by ImageJ (v1.38e), and it is calculated based on the following formula:

$$\begin{array}{l}{\rm{CTCF}}={\rm{integrated}}\; {\rm{density}}{{\mbox{--}}}\left(\right.{\rm{area}}\; {\rm{of}}\; {\rm{selected}}\; {\rm{cell}}\\ \qquad\qquad \times\; {\rm{mean}}\; {\rm{fluorescence}}\; {\rm{of}}\; {\rm{background}}\; {\rm{readings}}\left. \right).\end{array}$$

Co-localization analysis and quantification were performed by using Coloc2 and co-localization threshold plugins. In short, two channels were selected, and the region of interest was then highlighted by a selection/drawing tool in both channels to measure co-localization in the chosen region of interest. The value of volume percentage was used to calculate the co-localization of the two channels.

### Blood surface and intracellular staining (human study)

For surface staining, 100 µl of whole blood was stained with fluorochrome-conjugated antibodies in 50 μl of MACS buffer made of PBS containing, 2% FBS and 2 µM EDTA at room temperature (RT) for 30 min in the dark. Following staining, red blood cells were lysed with 2 ml of one-step fix/lyse solution for 20 min at RT, washed and resuspended with MACS buffer and analysed immediately. For intracellular staining, cells were fixed and permeabilized following the manufacturer’s instructions (BD Biosciences, 555028). Analysis was carried out with FlowJo (v10.6.2).

### In vitro human T cell activation, proliferation, IFN-γ production and glucose uptake

Freshly isolated PBMCs were plated in 0.25 × 10^6^per 200 µl of complete RPMI (Euroclone, ECM9006) or DMEM (Merck, D5030) at 2.75 mM, 5.5 mM or 11 mM glucose in 96-well U-bottom plates previously coated αCD3 (5 µg ml^−1^ Invitrogen, 14-0039-82) and αCD28 (2 µg ml^−1^, Invitrogen, 14-0289-82) in the presence of rhIL-2 (25 U ml^−1^, Roche, 10799068001) for 2 d or 6 d according to experimental purposes.

To measure T cell proliferation, PBMCs were stained with 5 μm of CFSE for 10 min at RT and washed three times in PBS/2% FBS/2 mM EDTA. After 6 d, cells were stained with human anti-CD4-BV605 (BD Biosciences, 565998) and anti-CD8-ef450 (Invitrogen, 48-0087-42).

For IFN-γ production, PBMCs were stimulated as described above for 2 d or 6 d, followed by a restimulation with soluble αCD3 (2.5 µg ml^−1^) and αCD28 (1 µg ml^−1^) in the presence of brefeldin (Merck, 555028) for 4 h. Cells were then stained with superficial human anti-CD4-BV605 and anti-CD8-ef450 and intracellular anti-IFN-γ-Af647, according to the manufacturer’s instructions (BD Biosciences, 557729).

To measure glucose uptake, PBMCs were washed in PBS and resuspended in glucose-free DMEM. A final concentration of 400 μM 6-NBDG was then added to the cells. Cells were incubated for an additional 15 min at 37 °C, washed twice with warm PBS, stained with anti-CD4 and CD8 fluorescent antibodies and immediately analysed by flow cytometry.

### Reporting summary

Further information on research design is available in the Nature Portfolio Reporting Summary linked to this article.

## Supplementary information

Supplementary Information Supplementary Methods and Tables 1 and 2

Reporting Summary

## Source data

Source Data Fig. 1 Statistical source data.

Source Data Fig. 2 Statistical source data.

Source Data Fig. 3 Statistical source data.

Source Data Fig. 4 Statistical source data.

Source Data Fig. 5 Statistical source data.

Source Data Fig. 6 Statistical source data.

Source Data Fig. 7 Statistical source data.

Source Data Fig. 8 Statistical source data.

Source Data Extended Data Fig. 1 Statistical source data.

Source Data Extended Data Fig. 2 Statistical source data.

Source Data Extended Data Fig. 3 Statistical source data.

Source Data Extended Data Fig. 4 Statistical source data.

Source Data Extended Data Fig. 5 Statistical source data.

Source Data Extended Data Fig. 6 Statistical source data.

Source Data Extended Data Fig. 7 Statistical source data.

## Data Availability

Source data are provided with this paper. All other original data (images) will be made available upon reasonable request.
