# Publisher Correction: The glucose transporter 2 regulates CD8+ T cell function via environment sensing

**DOI:** 10.1038/s42255-025-01256-3

**Published:** 2025-02-25

**Authors:** Hongmei Fu, Juho Vuononvirta, Silvia Fanti, Fabrizia Bonacina, Antonio D’Amati, Guosu Wang, Thanushiyan Poobalasingam, Maria Fankhaenel, Davide Lucchesi, Rachel Coleby, David Tarussio, Bernard Thorens, Robert J. Hearnden, M. Paula Longhi, Paul Grevitt, Madeeha H. Sheikh, Egle Solito, Susana A. Godinho, Michele Bombardieri, David M. Smith, Dianne Cooper, Asif J. Iqbal, Jeffrey C. Rathmell, Samuel Schaefer, Valle Morales, Katiuscia Bianchi, Giuseppe Danilo Norata, Federica M. Marelli-Berg

**Affiliations:** 1 William Harvey Research Institute, Faculty of Medicine and Dentistry, Queen Mary University of London, London, UK; 2 Department of Pharmacological and Biomolecular Sciences (DisFeB), Università Degli Studi di Milano, Milan, Italy; 3 Section of Anatomical Pathology Department of Precision and Regenerative Medicine, University of Bari Medical School, Bari, Italy; 4 Bart’s Cancer Institute, Faculty of Medicine and Dentistry, Queen Mary University of London, London, UK; 5 Faculty of Biology and Medicine, Center for Integrative Genomics, Génopode Building - UNIL Sorge, University of Lausanne, Lausanne, Switzerland; 6 Discovery Sciences, Innovative Medicines and Early Development Biotech Unit, AstraZeneca, Cambridge, UK; 7 Institute of Cardiovascular Sciences, University of Birmingham, Birmingham, UK; 8 Department of Pathology, Microbiology, and Immunology, Vanderbilt Center for Immunobiology, Vanderbilt University Medical Center, Nashville, TN USA

**Keywords:** Adaptive immunity, Cell biology, Metabolism

Correction to: *Nature Metabolism* 10.1038/s42255-023-00913-9, published online 26 October 2023.

In the version of this article initially published, due to a production processing error, the lower graphs in Fig. 1a, b, now labelled “CD4^+^,” appeared originally as “CD8^+^.” The figure has been amended in the HTML and PDF versions of the article.

