# Supplementary material for: The glucose transporter 2 regulates CD8+ T cell function via environment sensing
Source: Nat Metab. 2023 Oct 26;5(11):1969–85. doi: 10.1038/s42255-023-00913-9 (PMC10663157; doi:10.1038/s42255-023-00913-9)
Supplement: Supplementary file 1 — Supplementary Methods and Tables 1 and 2 [file 42255_2023_913_MOESM1_ESM.pdf]

nature metabolism

Article

https://doi.org/10.1038/s42255-023-00913-9

The glucose transporter 2 regulates CD8$^+$ T cell function via environment sensing

In the format provided by the authors and unedited

Supplementary Information

Supplementary Methods.

Characterization of patients from PLIC study. Genomic DNA was extracted using Flexigene DNA kit (Qiagen, Milan, Italy). Samples from the PLIC study were genotyped for the rs5400 missense mutation (G>A allelic change; https://www.ncbi.nlm.nih.gov/snp/rs5400) on the *SLC2A2* locus, by TaqMan-based allelic discrimination. 58 homozygous AA were found versus 209 GA heterozygous and 711 wild-type GG (Hardy-Weinberg, chi-squared= 51.189). The experimental analysis was conducted on a subgroup of 17 subjects, ten GG (*wild type*) and seven AA (*homozygous*) matched by age, sex and clinical and pharmacological history. Body Mass Index (BMI, Kg/m2) was calculated and the determination of plasma lipid profile, glucose levels, liver enzymes and whole blood leukocytes counts were available after an over-night fast (at least 10 hours) with a blood drawn from antecubital vein. Supplemental Table 1 reports these parameters.

Reagents

CFSE was purchased from Invitrogen and used at 5μM. PX-478 was purchased from Cayman chemical. Glucose-free T cell medium was purchased from MP Biomedicals. 6-NBDG and Pierce™ 16% Formaldehyde (w/v) were purchased from ThermoFisher. Dulbecco's Modified Eagle media (DMEM) was purchased from Merck. RPMI, glutamine, 2-Mercaptoethanol (2-ME), sodium pyruvate and HEPES were purchased from Gibco. Glucose, 2-Deoxy-D-glucose, oligomycin A, FCCP, rotenone, antimycin, red blood cell lysis buffer, DAPI (4',6-Diamidino-2-Phenylindole, Dihydrochloride) and dithiothreitol were purchased from Sigma-Aldrich; FBS was purchased from Seralab. Ficoll-PlaqueTM PREMIUM was purchased from GE-Healthcare. GolgiPlug was purchased from BD biosciences. High-Capacity RNA-to-cDNA™ Kit, ProLong Gold Antifade and LIVE/DEAD™ Fixable Aqua Dead Cell Stain Kit were purchased from Life Technologies. Intracellular Fixation & Permeabilization Buffer Set was

purchased from eBioscience. TaqMan human L 750 uL 80X – assay ID: C___2862880_1_ and TaqMan™ Genotyping Master Mix were purchased from Applied Biosystem. FlexiGene DNA kit (250 reactions) and RNeasy Mini Kit (50) were purchased from Qiagen. iQ™ SYBR® Green Supermix was purchased from Biorad. Murine IFNγ (clone H22), MojoSort™ Mouse CD8 T Cell Isolation Kit and MojoSort™ Mouse CD4 T Cell Isolation Kit were purchased from Biolegend. Mouse naive CD4 T cell isolation kit, Mouse CD4 T cell isolation kit and Mouse CD8 T cell isolation kit were purchased from STEMCELL. EasySep™ Human Naïve Pan T Cell Isolation Kit were purchased from STEMCELL. MojoSort™ Human CD8 T Cell Isolation Kit and MojoSort™ Human CD4 T Cell Isolation Kit were purchased from Biolegend. XF assay medium, Seahorse XF Cell Mito Stress Test Kit and Seahorse XF Glycolysis Stress Test Kit were purchased from Agilent Technologies. CD8 depletion antibody (clone 2.43) was purchased from BioXCell. Recombinant murine IP-10 (250-16) and recombinant murine IFNγ (315-05) were purchased from PeproTech. Recombinant murine Galectin-9 (3535-GA-050) was purchased from R&D SYSTEMS.

The Glut1 inhibitor STF-31 (SML1108) and the dual Glut1 and Glut2 inhibitor Phloretin (P7912) were purchased from Sigma Aldrich. Optimal doses were determined from available literature as well as titration followed by measurement of ECAR in activated T cells.

Cell surface and intracellular staining protocols.

For surface staining, cells were resuspended (10^7/ml) and stained with flurochrome-conjugated antibodies in 100μl of Flow cytometry buffer (PBS containing 1% FBS and 0.1% sodium azide) at 4°C for 30 minutes. CCR7 antibody staining was performed at 37°C for 30 minutes. Optimal antibody concentrations for staining were calculated based on manufacturer instructions. Following staining, cells were washed and resuspended with flow cytometry buffer and analyzed immediately. Alternatively, for delayed analysis, cells were fixed in fixation buffer (flow cytometry buffer containing 1% formaldehyde) for 30 minutes at 4°C, washed and stored in flow cytometry buffer at 4°C.

For stimulating cytokine production, T cells (2×10^6/ml) were re-stimulated with 50 ng/ml phorbol 12-myristate acetate (PMA) and 500 ng/ml ionomycin in the presence of 500µg/ml brefeldin A for at least 4 hours. Cells were first stained with antibodies for surface molecules. After washing, cells were fixed and permeabilized with intracellular fixation & permeabilization buffer for at least 30 minutes. Cells were then washed and resuspended in 1X perm solution containing antibodies for intracellular cytokines or isotype-matched control antibodies. After a final wash, the cells were resuspended in staining buffer for flow cytometry analysis.

For labelling T cells with intravital fluorescent probes, T cells were washed with PBS, counted and resuspended in PBS at a final concentration of 10^7/ml. Labeling of T cells with succinimidyl ester dyes CFSE was performed by incubating T cells in PBS containing final concentration of 5µm CFSE for 10-15 minutes at room temperature. The reaction was terminated by adding equal volume FBS and the cells were then washed with PBS.

Human PBMC isolation.

For each subject, 30 mL of blood (supplemented with EDTA) were split in two falcons of 15 mL and spin for 12 minutes at 1000xg. Plasma was discarded and the interface between plasma and red blood cells, enriched in leukocytes and platelets (buffy coat), was carefully collected, diluted with cold PBS and stratified on 3 mL of Ficoll-Plaque™ PREMIUM. After centrifugation of 35 minutes at 250xg, PBMC layer was carefully collected and was 3 times with 10 mL of cold PBS at 180xg for 12 minutes to get rid of platelets. PBMC were counted and used for subsequent analysis.

LC-MS/MS analysis. The liquid chromatography system was fitted with a Sequant ZIC-pHILIC column (150 mm × 2.1 mm) and guard column (20 mm × 2.1 mm) from Merck Millipore and the temperature maintained at 35°C. The sample (2 µL) was separated at a flow rate of 0.1 mL/min. The mobile phase was composed of 10 mM ammonium carbonate and 0.15% ammonium hydroxide in water (solvent A) and acetonitrile (solvent B). A linear gradient was applied by increasing the concentration of A from 20% to 80% within 22 minutes and then

maintained for 7 minutes. The mass spectrometer was operated in full MS and polarity switching mode, in the range of 70–1000 m/z and resolution 70,000. Major ESI source settings were: spray voltage 3.5 kV, capillary temperature 275°C, sheath gas 35, auxiliary gas 5, AGC target 3e6, and maximum injection time 200 ms. For the targeted analysis, the acquired spectra were analyzed using XCalibur Qual Browser and XCalibur Quan Browser software (Thermo Fisher Scientific). Compound discoverer 3.1 (Thermo Fisher Scientific) was used for untargeted and potentially novel feature detection and annotation with library scoring. Features with the fold change greater than 2 and $P < 0.05$ were selected as discriminating markers. Samples were analyzed by quadruplicate.

Supplementary Table 1. Biochemical characteristics of SLC2A2 (Glut2) wild type (GG) and homozygous SNP (AA) carriers

	**Wild type (GG)** (n=10)	**Homozygous (AA)** (n=7)
Total cholesterol (mg/dL)	178,80 (±34,28)	192,14 (±17,71)
HDL-C (mg/dL)	53,40 (±12,58)	59,71 (±14,38)
Triglycerides (mg/dL)	103,86 (±33,91)	101,80 (±25,02)
LDL-C (Friedewald)	111,57 (±19,74)	105,00 (±34,04)
Glucose (mg/dL)	120,20 (±38,37)	83,86 (±5,90) (*)
Insulin (U/ml)	6,11 (3,37-7,01)	6,03 (±3,64) (*)
Uric acid (mg/dL)	6,38 (±1,32)	4,31 (±1,17) (*)
Creatinine (mg/dL)	0,88 (0,71-1,05)	0,75 (±0,21)
ALT (U/l)	21,00 (13,50-24,00)	15,71 (±2,06)
AST (U/l)	24,00 (±7,33)	20,14 (±1,86)
GGT (U/l)	23,00 (13,75-31,00)	19,43 (±8,44)
CPK (mg/dL)	86,50 (50,00-161,00)	72,00 (58,00-79,00)
Leukocytes (cells* $10^3/\mu L$)	6,63 (±1,71)	7,33 (±2,62)
Hemoglobin (%)	14,14(±1,81)	13,76 (±1,24)
Hematocrit (%)	43,37 (±4,75)	42,37 (±4,10)
Platelets (/mm3)	233,30 (±55,39)	285,71 (±51,87)
Neutrophils (cells* $10^3/\mu L$)	3,93 (±1,16)	4,08 (±1,57)
Lymphocytes (cells*	1,85 (±0,70)	2,57 (±0,99) (*)
Monocytes (cells* $10^3/\mu L$)	0,59 (±0,13)	0,55 (±0,22)
Eosinophils (cells* $10^3/\mu L$)	0,22 (±0,09)	0,10 (±0,07)
Basophils (cells* $10^3/\mu L$)	0,04 (±0,02)	0,03 (±0,02)

Values are indicated as mean ± SD.

(*) indicates significantly altered values.

Supplementary Table 2. Primers used in the study.

Gene (protein)	Forward	Reverse
Tub1a (α-tubulin)	TCTCGCATCCACTTCCCTC	ATGCCCTCACCCACGTAC
Aco2 (Aconitase)	ATCGAGCGGGGAAAGACATAC	TGATGGTACAGCCACCTTAGG
Ogdh *(a-KG Dehydrogenase)*	TATGGCCTACACGAGTCTGAC	CCAGCCGACGGATGATCTC
Fh1 (Fumarase)	GAATGGCAAGCCAAAATTCCTT	CGTTCTGTAGCACCTCCAATCTT
Mdh1 (Malate dehydrogenase)	TTCTGGACGGTGTCCTGATG	TTTCACATTGGCTTTCAGTAGGT
Slc2a1 (Glut1)	CACTGTGGTGTCGCTGTTTG	ATGGAATAGGACCAGGGCCT
Slc2a2 (Glut2)	CCTACTTGGCCTATCTGCTGT	GCCCTGACTTCCTCTTCCAA
Slc2a3 (Glut3)	TCGGATGTCACAGGAGAAGC	CTGAGACAGCTGGAGGACAA
Slc2a6 (Glut6)	AACCGAGGGACTCGACTATGA	CAAGGCATACCCAAAGCTGAA
Slc2a8 (Glut8)	CCCTTCGTGACTGGCTTT	TGGGTAGGCGATTTCCGAGAT
Lgasl9 (Gal-9)	ATATCAACCTTCGCTGTGGAGG	CCCCAGGAGTTGTTGATCTG
Stomatin	CAGATTCAGCAACCCGTCTT	GTCCAGCGTACTCTGCATGTG
Hk1 (Hexokinase I)	TCACATTGTCTCCTGCATCTC	CTTTGAATCCCTTTGTCCACG
HKII (Hexokinase II)	TCAAAGAGAACAAGGGCGAG	AGGAAGCGGACATCACAATC
Pfkfb3	CTGACTCGCTACCTCAACTG	ACTGTTTTCGGACTCTCATGG
Pkm2	CCATTCTCTACCGTCCTGTTG	CCATGTAAGCGTTGTCCAG
Pdha1	ACATGGCTTCACCTTCACTC	CCGTTGCCTCCATAGAAGTTC
Cpt1a	CCAAGTATCTGGCAGTCGA	CGCCACAGGACACATAGT
Hif1a	TCTGAACGTCGAAAAGAAAAGTC	ACGTAAATAACTGATGGTGAGCC
Gzmb (Granzyme B)	CCACTCTCGACCCTACATGG	GGCCCCCAAAGTGACATTTATT
Ifng	TCAAGTGGCATAGATGTGGAAGAA	TGGCTCTGCAGGATTTTCATG
Tbx21 (T-bet)	CCTCTTCTATCCAACCAGTATC	CTCCGCTTCATAACTGTGT
