# Supplementary material for: The glucose transporter 2 regulates CD8+ T cell function via environment sensing
Source: Nat Metab. 2023 Oct 26;5(11):1969–85. doi: 10.1038/s42255-023-00913-9 (PMC10663157; doi:10.1038/s42255-023-00913-9)
Supplement: Supplementary file 2 — Reporting Summary [file 42255_2023_913_MOESM2_ESM.pdf]

nature portfolio

Corresponding author(s): Federica Marelli-Berg

Last updated by author(s): 24th August 2023

Reporting Summary

Nature Portfolio wishes to improve the reproducibility of the work that we publish. This form provides structure for consistency and transparency in reporting. For further information on Nature Portfolio policies, see our Editorial Policies and the Editorial Policy Checklist.

Please do not complete any field with "not applicable" or n/a. Refer to the help text for what text to use if an item is not relevant to your study.
For final submission: please carefully check your responses for accuracy; you will not be able to make changes later.

Statistics

For all statistical analyses, confirm that the following items are present in the figure legend, table legend, main text, or Methods section.

n/a	Confirmed	
☐	x	The exact sample size (n) for each experimental group/condition, given as a discrete number and unit of measurement
☐	x	A statement on whether measurements were taken from distinct samples or whether the same sample was measured repeatedly
☐	x	The statistical test(s) used AND whether they are one- or two-sided<br>Only common tests should be described solely by name; describe more complex techniques in the Methods section.
x	☐	A description of all covariates tested
☐	x	A description of any assumptions or corrections, such as tests of normality and adjustment for multiple comparisons
☐	x	A full description of the statistical parameters including central tendency (e.g. means) or other basic estimates (e.g. regression coefficient) AND variation (e.g. standard deviation) or associated estimates of uncertainty (e.g. confidence intervals)
☐	x	For null hypothesis testing, the test statistic (e.g. F, t, r) with confidence intervals, effect sizes, degrees of freedom and P value noted<br>Give P values as exact values whenever suitable.
x	☐	For Bayesian analysis, information on the choice of priors and Markov chain Monte Carlo settings
x	☐	For hierarchical and complex designs, identification of the appropriate level for tests and full reporting of outcomes
x	☐	Estimates of effect sizes (e.g. Cohen's d, Pearson's r), indicating how they were calculated

Our web collection on statistics for biologists contains articles on many of the points above.

Software and code

Policy information about availability of computer code

Data collection

Flow cytometry data were collected by CytExpert 2.4 of Cytoflex LX (Beckman). qPCR was performed by CFX-Connect RT-PCR System and CFX Manager Software Version2.1 (Bio-Rad). Seahorse was performed by Seahorse XFe96 Analyzer (Agilent). Metabolomics data was performed using a Q Exactive Quadrupole-Orbitrap mass spectrometer coupled to a Vanquish UHPLC system (Thermo Fisher Scientific). Glycogen test data were collected by SPECTROstar Omega (BMG LABTECH) using software V 5.11 R4. Electron microscopy data was collected by a JOEL JEM-1230 transmission electron microscope at an accelerating potential of 80 kV. Images were examined using Zeiss Z1 deconvolution microscope (Carl Zeiss) equipped with an AxioCam MRm cooled monochrome digital camera and an ApoTome.2 imaging unit. Some images were examined using a Leica SP5 confocal microscope. Confocal images and Z stacks were acquired and analyzed by Leica LAS software.

Data analysis

Flow cytometry data were analyzed by FlowJo (v10.6.2). Statistics and Data plotting were performed by GraphPad Prism (v9). OCR and ECAR were analyzed by seahorse wave software (Seahorse, Agilent Technologies. v2 .6). Quantification of metabolites was analyzed by XCalibur Qual Browser and XCalibur Quan Browser software (Thermo Fisher Scientific). Compound discoverer 3.1 (Thermo Fisher Scientific) was used for untargeted and potentially novel feature detection and annotation with library sc oring. Samples were analyzed by quadruplicate. Electron microscopy data were quantified and analysed via QuPath V0.43 and Graphpad 9 software. Microscopy imaging was analyzed by AxioVision Rel.4.8 and ImageJ 1.38e.

For manuscripts utilizing custom algorithms or software that are central to the research but not yet described in published literature, software must be made available to editors and reviewers. We strongly encourage code deposition in a community repository (e.g. GitHub). See the Nature Portfolio guidelines for submitting code & software for further information.

Data

Policy information about availability of data

All manuscripts must include a data availability statement. This statement should provide the following information, where applicable:

- Accession codes, unique identifiers, or web links for publicly available datasets
- A description of any restrictions on data availability
- For clinical datasets or third party data, please ensure that the statement adheres to our policy

Any additional materials and reagents are available from the corresponding author upon reasonable request. Source data are provided with this paper.

Research involving human participants, their data, or biological material

Policy information about studies with human participants or human data. See also policy information about sex, gender (identity/presentation), and sexual orientation and race, ethnicity and racism.

Reporting on sex and gender

The experimental subjects were matched by gender.

Reporting on race, ethnicity, or other socially relevant groupings

The experimental subjects were matched by age and race.

Population characteristics

The experimental subjects, wild type and matched age and sex carriers of SLC2A2, SNP were matched by clinical and pharmacological history. Body Mass Index (BMI, Kg/m2) was calculated and the determination of plasma lipid profile, glucose levels, liver enzymes and whole blood leukocytes counts were available after an over-night fast with blood drawn from antecubital vein.

Recruitment

The Progressione della Lesione Intimale Carotidea (PLIC) Study (a sub-study of the CHECK study) is a large survey of the general population of the northern area of Milan (n= 2.606), followed at the Center for the Study of Atherosclerosis, Bassini Hospital (Cinisello Balsamo, Milan, Italy). Genomic DNA was extracted and genotyped for missense mutation (G>A allelic change; https://www.ncbi.nlm.nih.gov/snp/rs5400) on the SLC2A2 locus, by TaqMan-based allelic discrimination. 58 homozygous AA were found versus 209 GA heterozygous and 711 wild-type GG (Hardy-Weinberg, chi-squared= 51.189). Participants were recruited according to matched age and sex without self-selection bias. The experimental analysis was conducted on a subgroup of 17 subjects, ten GG and seven AA

Ethics oversight

The Study was approved by the Scientific Committee of the Università degli Studi di Milano ("Cholesterol and Health: Education, Control and Knowledge – Studio CHECK ((SEFAP/Pr.0003) – reference number Fa-04-Feb-01) in February 4th 2001. An informed consent was obtained by subjects in accordance with the Declaration of Helsinki.

Note that full information on the approval of the study protocol must also be provided in the manuscript.

Field-specific reporting

Please select the one below that is the best fit for your research. If you are not sure, read the appropriate sections before making your selection.

[X] Life sciences [ ] Behavioural & social sciences [ ] Ecological, evolutionary & environmental sciences

For a reference copy of the document with all sections, see nature.com/documents/nr-reporting-summary-flat.pdf

Life sciences study design

All studies must disclose on these points even when the disclosure is negative.

Sample size

Sample sizes were based on common practice and our experience in the related fields, balancing resource availability, statistic robustness, and animal welfare. The sample size for each experiment is indicated in the figures and figure legends. No statistical methods were used to pre-determine sample sizes but our sample sizes are similar to those reported in previous publications1-3. For in vitro experiments, at least three independent sample were achieved. For in vivo experiments, n = 3-10 mice were used per experimental group.

Schwoebel, F., Barsig, J., Wendel, A. & Hamacher, J. Quantitative assessment of mouse skin transplant rejection using digital photography. *Lab Anim* **39**, 209-214 (2005).
Karkeni, E. *et al.* Vitamin D Controls Tumor Growth and CD8+ T Cell Infiltration in Breast Cancer. *Front Immunol* **10**, 1307 (2019).
Cao, Y., Rathmell, J.C. & Macintyre, A.N. Metabolic reprogramming towards aerobic glycolysis correlates with greater proliferative ability and resistance to metabolic inhibition in CD8 versus CD4 T cells. *PLoS One* **9**, e104104 (2014).

Data exclusions

No data was excluded from this study.

Replication

Biological replicates are performed to ensure reproducibility. For ex vivo and in vivo experiments, the number of replicates is equal to individual mice used. When representative data are shown, the experimental findings were reproduced independently at least twice with similar results.

Randomization

Randomization was applied wherever possible. For animal experiments, mice are sex and age matched and are randomly assigned to control and different treatment groups. For human subjects, participants were chosen on age and sex matched basis.

Blinding

For in vivo skin grafting and tumor experiments, data collection and analysis such as graft scoring and tumor measuring were performed by researchers who were blinded to group information. No blinding was involved in other experiments such as Seahorse assay, as machine-based readouts are not subject to investigator bias.

Reporting for specific materials, systems and methods

We require information from authors about some types of materials, experimental systems and methods used in many studies. Here, indicate whether each material, system or method listed is relevant to your study. If you are not sure if a list item applies to your research, read the appropriate section before selecting a response.

Materials & experimental systems

n/a	Involved in the study
[ ]	[x] Antibodies
[ ]	[x] Eukaryotic cell lines
[x]	[ ] Palaeontology and archaeology
[ ]	[x] Animals and other organisms
[ ]	[x] Clinical data
[x]	[ ] Dual use research of concern
[x]	[ ] Plants

Methods

n/a	Involved in the study
[x]	[ ] ChIP-seq
[ ]	[x] Flow cytometry
[x]	[ ] MRI-based neuroimaging

Antibodies

Antibodies used

For the phenotypic characterization of murine studies, cells were stained with: CD3-Alexa flour 700(clone 17A2, BioLegend, 100216); CD25 PE (clone PC61, BioLegend, 102008); CD26 PerCP/Cy5.5 (clone H194-112, eBioscience, **45-0261-82**); CD28 PE (clone 37.51, eBioscience, 12-0281-82); CD31 PE/Cy7 (clone 390, eBioscience, 12-0311-81); CD38 PE/Cy7 (clone 90, BioLegend, 102717); CD44 pacific blue ((clone IM7, BioLegend, 103020) or CD44 BV605 (clone IM7, BioLegend, 103047); CD45.1 FITC (clone A20, BioLegned, 110706); CD45.2 AF700 (clone 104, BioLegend, 109822); CD49d PE (clone 9C10, BioLegend, 103705); CD62L FITC (clone MEL-14, BioLegend, 104406); CD69 FITC (clone H1.2F3, eBioscience, 11-0691-82); Glut1 AF647 (clone EPR3915, abcam, ab195020) or AF 405 (polyclonal, NOVUS. NB110-39113AF405); Glut2 PE (clone 205115, NOVUS, FAB1440P); Gal-9 APC (clone 108A2, BioLegend, 137912) or PE/Cy7 (clone RG9-35, eBioscience, 25-9211-80); Stomatin AF488 (polyclonal, Bioss ANTIBODIES, bs-10443R-A488); MHCI (H-2kb/H-2Db) AF647 (clone 28-8-6, BioLegend, 114612); TCRβ BV605 (clone H57-597, BioLegend, 109241); CCR4 APC (clone 2G12, BioLegend, 131211); CCR5 PE (clone HM-CCR5, eBioscience, 12-1951-81); CCR6 PE (clone 29-2L17, BioLegend, 129804); CCR7 APC (clone 4B12, BioLegend, 120107); CXCR3 PerCP/Cy5.5 (clone CXCR3-173, BioLegend, 126514); CXCR4 FITC (clone 2B11/CXCR4, BD biosciences, 551967); LFA-1 PE/Cy7 (clone M17/4, BioLegend, 101122); B220 BV605 (clone RA3-6B2, BioLegend, 103243); CD107α PE/Dazzle 594 (clone 1D4B, BioLegend, 121624);

Phenotypic characterization of human cells was performed with: CD3 AF700 (clone UCHT1, Invitrogen, 56-0038-80); CD4 BV605 (clone SK3, also known as Leu3a, BD Biosciences, 565998); CD8 ef450 (clone SK1, BD Biosciences, 48-0087-42); CD8 FITC (clone RPA-T8, BioLegend, 301060); CCR7 FITC (clone REA546, Miltenyi Biotec, 130-120-468); CD45RA-BV785 (clone HI100); CD45RA-PerCPVio700 (clone REA1047, BioLegend, 304140); CD45RO- APCVio770 (clone REA611, Miltenyi biotec, 130-113-547); Glut2-PE (clone 199017, NOVUS, FAB1414P)

Intracellular staining for murine studies was performed with the following antibodies: FoxP3 APC (clone FJK-16s, eBioscience, 77-5775-40); Hif1α FITC (clone 241812, R&D, IC1935F) or Hif1α PE (clone 241812, R&D, IC1935P); Granzyme B FITC (clone GB11, BioLegend, 515403); IFNγ PerCP/Cy5.5 (clone XMG1.2, BioLegend, 505822); IL-17 ef450 (clone eBio17B7, eBioscience, 48-7177-82). IFNγ AF647 (clone B27, BD Biosciences, 557729) was used for intracellular cytokine staining in human cells.

Validation

Species and application of commercially available antibodies were validated by the company and researchers. An isotype control antibody were used at the same concentration to validate the antibody of interest.

Eukaryotic cell lines

Policy information about cell lines and Sex and Gender in Research

Cell line source(s)

E0771 breast adenocarcinoma cells (ABC-TC5564, Accegen Biotechnology), originally isolated from a spontaneous adenocarcinoma in C57BL/6 mice, were used as a syngeneic mouse model of breast cancer.

Authentication

E0771 breast adenocarcinoma cells are well described and authenticated by cell morphology and phenotyping. E0771 have been used and validated by previous researchers.

Mycoplasma contamination

E0771 cells were free of mycoplasma contamination.

Commonly misidentified lines
(See ICLAC register)

This line is not included in the ICLAC Register

Animals and other research organisms

Policy information about studies involving animals; ARRIVE guidelines recommended for reporting animal research, and Sex and Gender in Research

Laboratory animals

C57BL/6 and BALB/c mice were purchased from Charles River (UK). Ripglut1;glut2-/- mice (Referred to as Glut2-) were kindly provided by Bernard Thorens (University of Lausanne, Switzerland). B6.Kd (BL/6 transgenic for Kd) were a gift from Robert Lechler (King's College London). B6.129-Hif1atm3Rsjo/J (HIFaloxp) and B6.Cg-Tg(Cd4-cre)1Cwi/BfluJ mice were purchased from Jackson Laboratory. Galectin-9 knockout mice (B6(FVB)-Lgals9tm1.1Cfg/Mmucd) on a C57BL/6J background were obtained from the Mutant Mouse Resource and Research Centers, USA (originally deposited by J Paulson, The Scripps Research Institute, USA). Female Marilyn mice, bearing a transgenic TCR specific for the male minor transplantation antigen HY peptide epitope Dby and restricted by H2-Ab molecules, have been previously described. Mata Hari mice, bearing a transgenic TCR specific for the male minor transplantation antigen HY peptide epitope Uty and restricted by H2-Db molecules, have been previously described.
Most experiments used mice at the age between 8 and 12 weeks, with exception of chimera mice at around 4 months old.

Wild animals

No wild animal were used in this study

Reporting on sex

For in vitro experiments, we used both female and male mice, and the results were not affected by sex of mice. For in vivo T cells adoptive transfer experiments, only female sex were used for donor Marilyn and Mata Hari mice and recipient mice due to the nature of male antigen specific transgenes. Also only female mice were used for tumor experiments as E0771 cells were used as a mouse model of breast cancer.

Field-collected samples

No field-collected samples were used in this study

Ethics oversight

All animal experiments were approved by Queen Mary Ethics of Research Committee in Queen Mary University of London (Establishment licence number: XEDA0F7B1), and conducted with strict adherence to the Home Office guidelines (PPL P71E91C8E).

Flow Cytometry

Plots

Confirm that:

[X] The axis labels state the marker and fluorochrome used (e.g. CD4-FITC).

[X] The axis scales are clearly visible. Include numbers along axes only for bottom left plot of group (a 'group' is an analysis of identical markers).

[X] All plots are contour plots with outliers or pseudocolor plots.

[X] A numerical value for number of cells or percentage (with statistics) is provided.

Methodology

Sample preparation

Tissues from spleen, lymph nodes and tumor were mashed and passed through 70 micron cell strainers. For surface marker staining, cells were suspended in FACS buffer (PBS with 2% FBS) and stained with Live/Dead Fixable Aqua staining kit abd fluorescently conjugated antibodies. For detecting cytokine production, cells were stimulated with 50 ng/ml phorbol 12-myristate

(PMA) and 500 ng/ml ionomycin in the presence of 500μg/ml brefeldin A for at least 4 hours. Cells were stained for surface markers and then fixed/permeabilized for staining with cytokines antibodies in permeabilization buffer. After staining, cells were suspended in FACS buffer for flow cytometry.

Instrument

Beckman Cytoflex LX

Software

CytExpert 2.4 was used to collect data. FlowJo (v10.6.2) was used to analyze the flow cytometry data.

Cell population abundance

Cell population abundance varied from 53% for CD4 T cells and 37% for CD8 T cells to 13.4% for IFNγ+CD8+ T cells

Gating strategy

Total cells were first gated on a forward scatter /side scatter plot and then gated on Aqua-low live cells. Single cells were identified using forward scatter width (FSC-W) and forward scatter area (FSC-A) to analyze cell size. These were then further identified for subsets of myeloid (CD45high/CD11b high) and lymphoid (CD45high/CD11b low). Myeloid sunset were then separated into populations of DCs (CD11c+), monocyte (Ly6C high/Ly6G low), neutrophil (Ly6C high/Ly6G high), and macrophage (F4/80 high/Ly6C low/Ly6G low). Lymphoid subset were then further phenotyped into B cells according to CD19 expression and T cells based on CD3 expression, which were further identified as CD8+ or CD4+ T cell population. NK cells were identified as NK1.1 positive.

[X] Tick this box to confirm that a figure exemplifying the gating strategy is provided in the Supplementary Information.
